# Biotic and Abiotic Constraints in Mungbean Production—Progress in Genetic Improvement

**DOI:** 10.3389/fpls.2019.01340

**Published:** 2019-10-25

**Authors:** Ramakrishnan M. Nair, Abhay K. Pandey, Abdul R. War, Bindumadhava Hanumantharao, Tun Shwe, AKMM Alam, Aditya Pratap, Shahid R. Malik, Rael Karimi, Emmanuel K. Mbeyagala, Colin A. Douglas, Jagadish Rane, Roland Schafleitner

**Affiliations:** ^1^World Vegetable Center, South Asia, Hyderabad, India; ^2^Myanmar Department of Agricultural Research, Nay Pyi Taw, Myanmar; ^3^Pulses Research Centre, Bangladesh Agricultural Research Institute (BARI), Gazipur, Bangladesh; ^4^Crop Improvement Division, ICAR-Indian Institute of Pulses Research (IIPR), Kanpur, India; ^5^Pakistan Agricultural Research Council, Islamabad, Pakistan; ^6^Kenya Agricultural and Livestock Research Organization (KALRO), Katumani, Kenya; ^7^National Agricultural Research Organization-National Semi-Arid Resources Research Institute (NARO-NaSARRI), Soroti, Uganda; ^8^Agri-Science Queensland, Department of Agriculture and Fisheries, Hermitage Research Facility, Warwick, QLD, Australia; ^9^National Institute of Abiotic Stress Management, Baramati, India; ^10^World Vegetable Center, Tainan, Taiwan

**Keywords:** mungbean, breeding, stresses, insect-pests, diseases, marker-assisted selection

## Abstract

Mungbean [*Vigna radiata* (L.) R. Wilczek var. *radiata*] is an important food and cash legume crop in Asia. Development of short duration varieties has paved the way for the expansion of mungbean into other regions such as Sub-Saharan Africa and South America. Mungbean productivity is constrained by biotic and abiotic factors. Bruchids, whitefly, thrips, stem fly, aphids, and pod borers are the major insect-pests. The major diseases of mungbean are yellow mosaic, anthracnose, powdery mildew, Cercospora leaf spot, halo blight, bacterial leaf spot, and tan spot. Key abiotic stresses affecting mungbean production are drought, waterlogging, salinity, and heat stress. Mungbean breeding has been critical in developing varieties with resistance to biotic and abiotic factors, but there are many constraints still to address that include the precise and accurate identification of resistance source(s) for some of the traits and the traits conferred by multi genes. Latest technologies in phenotyping, genomics, proteomics, and metabolomics could be of great help to understand insect/pathogen-plant, plant-environment interactions and the key components responsible for resistance to biotic and abiotic stresses. This review discusses current biotic and abiotic constraints in mungbean production and the challenges in genetic improvement.

## Introduction

Mungbean [*Vigna radiata* (L.) R. Wilczek var. *radiata*] is a short-duration grain legume cultivated over 7 million hectares, predominantly across Asia and rapidly spreading to other parts of the world. Mungbean seeds are rich in proteins (∼24% easily digestible protein), fiber, antioxidants, and phytonutrients (Itoh et al., 2006). Mungbean is consumed as whole seed or split cooking, flour, or as sprouts, thus, forms an important source of dietary protein. Mungbean sprouts contain high amounts of thiamine, niacin, and ascorbic acid. Yield potential of mungbean is in the range of 2.5–3.0 t/ha, however, the average productivity of mungbean is staggering low at 0.5 t/ha. The low productivity is due to abiotic and biotic constraints, poor crop management practices and non-availability of quality seeds of improved varieties to farmers (Chauhan et al., 2010; Pratap et al., 2019a). The major biotic factors include diseases such as yellow mosaic, anthracnose, powdery mildew, Cercospora leaf spot (CLS), dry root rot, halo blight, and tan spot, and insect-pests especially bruchids, whitefly, thrips, aphids, and pod borers (Lal, 1987; Singh et al., 2000; War et al., 2017; Pandey et al., 2018). Abiotic stresses affecting mungbean production include waterlogging, salinity, heat, and drought stress (HanumanthaRao et al., 2016; Singh and Singh, 2011). Genetic diversity in cultivated mungbean is limited due to breeding efforts that were restricted to relatively few parental lines and hence the need to broaden the narrow genetic base of cultivated mungbeans. Development of short-duration varieties has paved the way for expansion of mungbean into different cropping systems (rice–rice, rice–wheat and rice-maize intercropping) and for cultivation in other regions of the world including Sub-Saharan Africa and South America (Shanmugasundaram, 2007; Moghadam et al., 2011). In order to improve productivity and stabilize crop production, there is a need to develop varieties resistant to biotic and abiotic stress factors. Breeding information on the biotic and abiotic stresses in mungbean and on the influence of environmental stresses at different plant development stages is essential to identify the sources for tolerance traits expressed at the right stage. With advanced technologies *viz*., phenotyping, genomics, proteomics and metabolomics, the genetic basis of plant interactions with pest, pathogen, and environment can be dissected to design effective crop improvement strategies. In this context, we discuss the biotic and abiotic constraints in mungbean, and the breeding efforts to improve this short duration crop.

## Biotic Stress in Mungbean

### Major Diseases and Economic Impacts

Viral, bacterial, and fungal diseases are of economic importance in South Asia, South East Asia, and Sub-Saharan Africa (Taylor et al., 1996; Singh et al., 2000; Raguchander et al., 2005; Mbeyagala et al., 2017; Pandey et al., 2018). Mungbean yellow mosaic disease (MYMD) is an important viral disease of mungbean (Singh et al., 2000; Noble et al., 2019). MYMD is caused by several begomoviruses, which are transmitted by whitefly *Bemisia tabaci* (Gennadius) (Hemiptera: Aleyrodidae) (Nair et al., 2017). The major fungal diseases are Cercospora leaf spot (CLS) [*Cercospora canescens* Ellis & G. Martin], powdery mildew (*Podosphaera fusca* (Fr.) U. Braun & Shishkoff, *Erysiphe polygoni* (Vaňha) Weltzien) and anthracnose (*Colletotrichum acutatum* (J.H. Simmonds), *C. truncatum* (Schwein.) Andrus & Moore, *C. gloeosporioides* (Penz.) Penz. & Sacc). Dry root rot [*Macrophomina phaseolina* (Tassi) Goid] is an emerging disease of mungbean. The less important ones are web blight (*Rhizoctonia solani* Kuhn), Fusarium wilt (*Fusarium solani* (Mart.) Sacc) and Alternaria leaf spot (*Alternaria alternata* (Fr.) Keissl) (Ryley and Tatnell, 2011; Pandey et al., 2018). Halo blight (*Pseudomonas syringae* pv. *phaseolicola*), bacterial leaf spot (*Xanthomonas campestris* pv. *phaseoli*), and tan spot (*Curtobacterium flaccumfaciens* pv. *flaccumfaciens*) are the important bacterial diseases. The economic losses due to MYMD account for up to 85% yield reduction in India (Karthikeyan et al., 2014). Dry root rot caused 10–44% yield losses in mungbean production in India and Pakistan (Kaushik and Chand, 1987; Bashir and Malik, 1988). Reports of yield losses of 33–44% due to Rhizoctonia root rot (Singh et al., 2013a) and 30–70% due to anthracnose (Kulkarni, 2009; Shukla et al., 2014) from India were estimated. Yield losses due to CLS were 97% in Pakistan and different states of India (Iqbal et al., 1995; Chand et al., 2012; Bhat et al., 2014), and 40% due to powdery mildew (Khajudparn et al., 2007). Among the minor fungal diseases, 20% yield loss was reported due to Fusarium wilt (Anderson, 1985) and 10% due to Alternaria leaf spot (Maheshwari and Krishna, 2013). A survey of mungbean fields throughout China between 2009–2014 reported average yield reductions of 30–50% and total crop failure in severely infected fields due to halo blight (Sun et al., 2017). Halo blight is an emerging disease in China (Sun et al., 2017) and Australia (Noble et al., 2019). In Iran, 70% incidence (Osdaghi, 2014) and in India 30% incidence (Kumar and Doshi, 2016) of bacterial leaf spot (*X. phaseoli*) has been reported. Studies were carried out to investigate the efficacy of bactericides, fungicides, bio-fungicides and botanicals in seed treatment and foliar spray and impact of cultural practices to reduce mungbean diseases (Pandey et al., 2018). Deployment of varieties with genetic resistance is the most effective and durable method for integrated disease management.

## Breeding for Resistance to Viral Diseases

Research into resistance to MYMD has been underway since 1980, with mutant genotypes developed from local germplasm by mutation breeding (gamma irradiation) at the National Institute for Agriculture and Biology, Pakistan, which later led to the development of the popular NM series varieties including NM 92 and NM 94 (Ali et al., 1997). Researchers reported that in mungbean, the genetic resistance against MYMD is governed by a single recessive gene (Reddy, 2009a), a dominant gene (Sandhu et al., 1985), two recessive genes and complementary recessive genes (Pal et al., 1991; Ammavasai et al., 2004). The mungbean variety NM 92 showed a resistant reaction against MYMD due to a single recessive gene (Khattak et al., 2000). Dhole and Reddy (2012) reported that two recessive genes governed the segregation ratio in the F_2_ population in six crosses between resistant and susceptible genotypes. However, F_2_ and F_3_ populations developed through an inter-specific [TNAU RED × VRM (Gg) 1] and intra-specific [KMG 189 × VBN (Gg)] crosses showed role of a single recessive gene in MYMD resistance (Sudha et al., 2013). Saleem et al. (1998) in their study with F_2_ populations derived from crosses between two local lines (NM-92 and NM-93-resistant to MYMD) and four exotic lines (VC-1973A, VC-2254A, VC-2771A and VC-3726A-susceptible to MYMD), found that susceptibility and resistance were controlled by a single genetic factor and that susceptibility was dominant over resistance. Similar results were recorded by Jain et al. (2013) in F_2_ and F_3_ populations of crosses between five susceptible (LGG 478, KM6 202, PUSA 9871, K 851, and KM6 204) and 4 resistant (KM6201, Sonamung, Samrat, and KM6 220) lines, and it was reported that the inheritance was governed by single dominant gene. However, two recessive genes were found to be responsible for MYMD resistance in the populations developed from crosses between two resistant (Satya and ML 818) and two susceptible (Kopergoan and SML 32) cultivars (Singh et al., 2013b). However, in the study of Mahalingam et al. (2018) two dominant genes governed MYMD resistance in the crosses between resistant (SML 1815, MH 421) and susceptible [VBN (Gg) 3, VBN (Gg) 2, LGG 460, RMG 10-28, and TM 96-2] genotypes. The major genes controlling MYMD resistance in the two crosses (KPSI × BM 6 and BM1 × BM 6) using six (P_1_, P_2_, F_1_, F_2_, BC_1_, and BC_2_) generations were estimated within 1.63–1.75 loci (Alam et al., 2014)

It is important to identify the strain/species of the virus causing the disease to make comparison between the different studies done. In repeated samplings over consecutive years in India, Nair et al. (2017) reported genetic similarity of MYMV strains from mungbean to a strain from Urdbean [*Vigna mungo* (L.) Hepper] (MYMV-Urdbean) dominant in North India, strains most similar to MYMV-*Vigna* predominant in South India, and *Mungbean yellow mosaic India virus* (MYMIV) strains predominant in Eastern India. The resistance sources of mungbean genotypes to MYMD (**Table 1**) can be used as potential donors and to develop mapping populations for the development of potential markers for MYMD. For the development of resistant lines, researchers have deployed plant-breeding methods with traditional methods of disease screening. In this regard, marker-assisted selection (MAS) is the most promising technique for disease resistant cultivar development. The study of genotypic diversity and the discovery of linked markers for *R* gene and quantitative trait loci (QTL) maps construction through molecular markers has improved the adeptness in the breeding programs conferring resistance for MYMD (Sudha et al., 2013). Basak et al. (2004) developed a yellow mosaic virus resistance linked marker named ‘VMYR1’ in mungbean. Among the parents, one pair, resistance gene analog (RGA) 1F-CG/RGA 1R (445bp DNA) of gene was found to be polymorphic out of 24 pairs of RGA primers screened. In F_2_ and F_3_ families, the polymorphisms were found to be linked with YMV-reaction. Binyamin et al. (2015) used sequence characterized amplified region-based markers linked with the MYMD-resistance gene for the screening of mungbean genotypes against the disease. In the resistant and tolerant genotypes, marker amplified desired bands were reported, while no amplification was observed in susceptible genotypes. Maiti et al. (2011) identified two MYMD-resistance marker loci, *CYR1* and *YR4* completely linked with MYMD-resistant germplasms and co-segregating with MYMD-resistant F_2_ and F_3_ progenies. Holeyachi and Savithramma (2013) identified random amplified polymorphic DNA (RAPD) markers linked with MYMD recombinant breeding lines. They reported that out of 20 random decamers, only 10 primers showed polymorphism between parents China mung (S) and BL 849 (R) and among them, only one primer (UBC 499) amplified a single 700 bp band in the resistant parent (BL 849) that was absent in susceptible genotype (China mung). Kalaria et al. (2014) studied the polymorphism by using 200 RAPD and 17 inter simple sequence repeat (ISSR) markers. Among RAPD markers, OPJ-18, OPG-5, and OPM-20 and in ISSR DE-16 were found to be potential ones, as they produced 28, 35, 28, and 61 amplicons, respectively. The resistant genotypes NAUMR1, NAUMR2, NAUMR3, and Meha were clearly separated from the susceptible cultivar, GM4. In another study, 5 QTLs based on simple sequence repeats (SSR) markers were investigated against MYMD, of them, three were from India (*qYMIV1, qYMIV2*, and *qYMIV3*) and 2 were from Pakistan (*qYMIV4* and *qYMIV5*) (Kitsanachandee et al., 2013). The QTL, *qYMIV1* explained 9.33% variation in disease response. Similarly, *qYMIV2* explained 10.61%, *qYMIV3* explained 12.55%, *qYMIV4* explained 21.55% and *qYMIV5* explained 6.24% variations in the disease response. Two major QTLs controlling genes on linkage group 2 (*qMYMIV2*) and 7 (*qMYMIV7*) resistant to MYMD were reported. These QTLs were conferring resistance in both F_2_ and BC_1_F_1_ populations with a coefficient of determination (R^2^) of 31.42–37.60 and 29.07–47.36%, respectively (Alam et al., 2014). Markers linked to QTLs in this study will be useful in marker-assisted breeding for the development of MYMD resistant mungbean varieties. During the growing season plant breeders can conduct repeated genotyping in the absence of disease incidence by applying linked marker-assisted genotyping. This technique will save labor and time during the introgression of MYMD-resistance through molecular breeding, as phenotyping against begomoviruses is complex, labor and time consuming. New donors of MYMD resistance have also been identified from interspecific sources (Chen et al., 2012; Nair et al., 2017).

**Table 1:** Resistant sources of mungbean against mungbean yellow mosaic disease.

Genotype(S)	Resistant level*	Country	References
NM-10-12-01	R	Thailand	Akhtar et al. (2009)
NM-2, VC-3960 (A-88), 98-CMH-016, VC-3960 (A-89), BRM-195	R	Pakistan	Bashir et al. (2005)
014043, 014133, 014249, 014250	R	Pakistan	Iqbal et al. (2011)
08	MR		
ML 1265, ML 1229	R	India	Kooner and Cheema (2007)
SML 1815, MH 421	R	India	Mahalingam et al. (2018)
BPMR-145, Vaibhav, Phule M-2003-3, TARM-18, Phule M-2002-13, Phule M-2001-3, Phule M-2002-17, Phule M-2001	R	India	Mandhare and Suryawanshi (2008)
EC300072, K141	R	India	Manivannan et al. (2001)
LGG424B, LM108B	I		
VC-6960-88, VC-6773 (B-G), VC-3960-89, ACC-12840014, VC-1089 A	R	Bangladesh	Mondol et al. (2013)
NCM-15-11, AZRI-1, AZRI-06, NCM-21, 14063, NCM-11-8	R	Pakistan	Munawar et al. (2011)
NM 94	T- Odisha and Andhra Pradesh MR- Tirunelveli	India	Nair et al. (2017)
ML1628	T		
VRMG(g)1, LM 235 (GY), K 851, T 44, Nelambur, Sona Moong, AVRDC 1785/5, LM 150, Madura moong, TNAU 26, WBM 202 (GY), KM 2, TARM 22, HUM 1, LGG 429/1, TARM2/2, TARM2/1, NM 94, Bari mung 2	R	India	Pandiyan et al. (2007)
ML267, LGG407	R	India	Panduranga et al. (2011)
ML-5, ML 405, ML 408, ML 337, MUM 2, VGG3 45, Pusa 8773	R	India	Patel and Srivastava (1990)
ML-818	R	India	Paul et al. (2013)
ML-9	MR		
GG-89 and GG-39, R: TM-98-50, TM-97-55, Co-5	I	India	Salam et al. (2009)
IPM 2-14, PDM139	R	India	Suman et al. (2015)
HUM 1, HUM 12, DMS 03-17-2, Pant Mung 4, Pusa 9531, HUM 16, Meha, RMG 62, TMB 37	MR		
ML-881, UPM-98,	HR	India	Yadav and Dahiya (2004)
Ganga-14, HUM-I, PDM-262, HUM-8	R		

*(T, Tolerant; I, Immune; HR, Highly resistant; R, Resistant; MR, Moderately resistant).

## Breeding for Resistance to Fungal Diseases

Researchers screened mungbean genotypes against fungal diseases from different countries in controlled and field conditions in order to identify sources of resistance. Resistant genotypes reported by investigators against various fungal diseases are presented in **Table 2**. It may be noted that screening of mungbean genotypes against powdery mildew and Cercospora leaf spot diseases has been much explored. However, little work has been done on the identification of sources of resistance against anthracnose and dry root rot and needs to be addressed as future priorities. Screening of mungbean genotypes against fungal diseases provided in **Table 2** were carried out under natural conditions, except for dry root rot, Khan and Shuaib (2007) screened in laboratory conditions.

**Table 2:** Resistant genotypes of mungbean against fungal diseases.

Diseases	Genotype(s)	Resistant level*	Country	References
**Anthracnose Cercospora leaf spot (CLS)**	ML1464, ML1486, ML1194 and ML1349	R	India	Kaur et al. (2011)
	V1471, V2773, V2757, V5036 and V4718	R	Taiwan	Hartman et al. (1993)
	M5-22 and M5-25	R	Thailand	Wongpiyasatid et al. (1999)
	BRM-188, C2/94-4-42, NM-98, 98-cmg-003, NM-1, NM-2, 98cmg-018, Basanti, PDM-11, CO-3, BARIMung-2 and VC3960-88	HR	Pakistan	Iqbal et al. (2004)
	ML5, 453, 443, 515, 611, 610, 613, 682, 713, 688, 735, 728, 746, 759 and 769	R	India	Singh et al. (2004)
	PANT M103, PUSA 105, PANT M3, PANT M2, ML 613, ML 173, ML 561, ML 347, PDM 11 and PANT M4	R	India	Marappa (2008)
	ML1464, ML1486, ML1194 and ML1349	R	India	Kaur et al. (2011)
	GM-02-08, GM-03-03 and GM-02-13	R	India	Yadav et al. (2014b)
	LGG-460	HR		
	ML-5, HUM-9, ML-4, HUM-4, SM-9-124, HUM-1, LGG-450, and SM-9-107	R	India	Singh and Singh (2014)
	1224-52 and 12404	HR	India	Zhimo et al. (2013)
	AKM 9910, ML 1299, IPM 02-5, and SML 668	R	India	Akhtar et al. (2014)
	KMP-13	MR	India	Bhaskar (2017)
**Powdery mildew**	V4189, V2159, V4207, V4668, V4990 and V4574	R	Taiwan	Hartman et al. (1993)
	V3912 and V4186	R/HR		
	V1104, V4658, V4631, V4717, V4662, and V4883	HR		
	M5-10 and M5-25	R	Thailand	Wongpiyasatid et al. (1999)
	BPMR-145, TARM-18, Vaibhav, Phule M-2002-13, Phule M-2003-3, Phule M-2001-3, Phule M-2001-5 and Phule M-2002-17	R	India	Mandhare and Suryawanshi (2008)
	TARM-18	R	India	Sujatha et al. (2011)
	LGG-460	R	India	Yadav et al. (2014a)
	BL 849, BL 865, LM1668, PMB 63 and AKM 8803	HR	India	Ramakrishnan and Savithramma (2014)
	KGS 83, Pusa 572, MH 96-1, GS 33-5, GS 21-5, AKM 99-4, COGG 936, TMB 47, ML 1299, MH 429, HUM 1, MH 429 and MH 530	HR	India	Akhtar et al. (2014)
	C1-34-23, C1-32-22, C1-37-23, C1-28-20, C1-38-27, C1-44-31, C1-175-111, C1-41-28, C1-246-159, C1-236-152, C1-275-177	HR	India	Kumar et al. (2017)
	KMP-36, KMP39 and KMP41	HR	India	Bhaskar (2017)
**Macrophomina blight** **Dry root rot (DRR)**	ML1464, ML1486, ML1194 and ML1349	R	India	Kaur et al. (2011)
	MSJ 118, KM 4-59 and KM 4-44	R	India	Choudhary et al. (2011)
	40504, 40457, NCM 257-5, 6368-64-72 and NCM 251-4	R	Pakistan	Khan and Shuaib (2007)
	NCM 252-10 and 40536	HR		

*HR, Highly resistant; R, Resistant; MR, Moderately resistant; adopted from Pandey et al. (2018).

Efficient breeding for fungal stresses requires readily available resistant germplasm and markers linked with QTL regions or major genes that can be employed in marker-assisted selection (MAS). In mungbean, for Cercospora leaf spot and powdery mildew molecular markers have been identified for application in breeding programs. However, QTLs or molecular markers for dry root rot and anthracnose have not been investigated. Both qualitative and quantitative modes of inheritance have been reported for resistance to powdery mildew Kasettranan et al. (2009). Single dominant gene control of resistance to powdery mildew was reported (AVRDC, 1979; Khajudparn et al., 2007; Reddy, 2009b), while Reddy et al. (1994) reported that two major dominant genes control the resistance. Chaitieng et al. (2002) and Humphry et al. (2003) found that one QTL conferred the resistance to powdery mildew, while Young et al. (1993) reported three QTLs linked with powdery mildew resistance. Young et al. (1993) made the conclusion from studying the mapping population developed from mungbean line VC3890 as a resistance parent. The population developed from a cross between KPS 2 (moderately resistant) and VC 6468-11-1A (resistant) mungbean genotypes was investigated by Sorajjapinun et al. (2005) and they reported additive gene action control of resistance. Kasettranan et al. (2010) identified SSR markers based QTLs such as *qPMR-1* and *qPMR-2* associated with resistance to powdery mildew. One major QTL on the linkage group 9 and two minor QTLs on linkage group 4 were identified in mungbean line V4718 (Chankaew et al., 2013). The mapping population against powdery mildew developed from mungbean line RUM5 resulted in two major QTLs on LG6 and LG9 and one minor QTL on LG4 (Chankaew et al., 2013). Fine mapping with populations developed from crosses between highly susceptible and highly resistant parents would be reliable for the identification of reliable markers.

Lee (1980) reported that a single dominant gene governs the resistance to CLS. Reports on quantitative genetic control of resistance to CLS (Chankaew et al., 2011) and a single recessive gene control (Mishra et al., 1988) have been reported. One major QTL (*qCLS*) for CLS located on linkage group 3, which explained 66-81% phenotypic variation was reported (Chankaew et al., 2011) using F_2_ (CLS susceptible cultivar Kamphaeng Saen1, KPS1 × CLS-resistance mungbean line, V4718) and BC_1_F_1_ [(KPS1 × V4718) × KPS1] populations.

## Breeding for Resistance to Bacterial Diseases

Bacterial pathogens are seed-borne and can persist in crop residue. Varietal resistance is recognized as the cornerstone of integrated disease management (Noble et al., 2019). Little work has been done on the screening of mungbean genotypes against bacterial diseases and identifying genetic markers associated with bacterial diseases in mungbean. From India, Patel and Jindal (1972) evaluated 2160 genotypes of mungbean for resistance to bacterial leaf spot (*X. phaseoli*) and reported that Jalgaon 781, P 646, P 475, and PLM 501 mungbean genotypes were resistant. From Pakistan, 8 out of 100 mungbean genotypes, were reported as resistant against bacterial leaf spot disease under field conditions (Iqbal et al., 1991; Iqbal et al., 2003). Munawar et al. (2011) screened 51 genotypes against bacterial leaf spot disease in Pakistan, and found NCM11-8, NCM 15-11, AZRI-1, and 14063 mungbean genotypes as resistant in natural incidence of the disease. In their field evaluation, few genotypes such as NCM 258-10, NCM-21, NCM 11-6, AZRI-06, and NCM 11-3 showed moderate resistance reaction.

The inheritance of bacterial leaf blight is governed by a single dominant gene (Thakur et al., 1977). Patel and Jindal (1972) reported that in mungbean genotypes Jalgaon 781, P 646, P 475, and PLM 501, the inheritance of resistance to bacterial leaf blight (BLB) was monogenic dominant. While QTLs were identified for bacterial leaf blight disease in other crops like chickpea (Dinesh et al., 2016), no records are available on QTLs of mungbean against bacterial disease. Screening for halo blight and tan spot has been carried out by the Australian breeding program in both controlled (glasshouse) and field conditions to identify useful donors as well as resistant progenies (Noble et al., 2019). Identification of genetic markers/QTLs associated with halo blight, tan spot, and bacterial leaf spot disease resistance in mungbean will accelerate the development of resistant commercial cultivars. These markers can be established through genome-wide association studies using large, diverse mungbean mapping populations’ representative of worldwide germplasm (Schafleitner et al., 2015; Noble et al., 2019).

## Major Insect-Pests and Economic Impacts

Insect-pests attack mungbean at all crop stages from sowing to storage and take a heavy toll on crop yield. Some insect-pests directly damage the crop, while others act as vectors of diseases. The economically important insect-pests in mungbean include stem fly, thrips, aphids, whitefly, pod borer complex, pod bugs, and bruchids (Swaminathan et al., 2012). Stem fly (bean fly), *Ophiomyia phaseoli* (Tryon), is one of the major pests of mungbean. Other species of stem fly that infest mungbean include *Melanagromyzasojae* (Zehntner) and *Ophiomyia centrosematis* (de Meijere) (Talekar, 1990). This pest infests the crop within a week after germination and under epidemic conditions, it can cause total crop loss (Chiang and Talekar, 1980). Whitefly, *B. tabaci* is a serious pest in mungbean and damages the crop either directly by feeding on phloem sap and excreting honeydew on the plant that forms black sooty mould or indirectly by transmitting MYMD. Whitefly’s latent period is less than four hours and a single viruliferous adult can transmit the MYMV within 24 h of acquisition and inoculation. The male and female whiteflies can retain the infectivity of the virus for 10 and 3 days, respectively. Further, *B. tabaci* complex consists of 34 cryptic species (Boykin and De Barro, 2014). Whitefly causes yield losses between 17 and 71% in mungbean (Marimuthu et al., 1981; Chhabra and Kooner, 1998; Mansoor-Ul-Hassan et al., 1998). Thrips infest mungbean both in the seedling and in flowering stages. The seedling thrips are *Thrips palmi* Karny and *Thrips tabaci* Lindeman and the flowering thrips are *Caliothrips indicus* Bagnall or *Megalurothrips* spp. During the seedling stage, thrips infest the seedling’s growing point when it emerges from the ground, and under severe infestation, the seedlings fail to grow. Flowering thrips cause heavy damage and attack during flowering and pod formation. They feed on the pedicles and stigma of flowers. Under severe infestation, flowers drop and no pod formation takes place. Spotted pod borer, *Maruca vitrata* (Fab.) is a major insect-pest of mungbean in the tropics and subtropics. With an extensive host range and distribution, it is widely distributed in Asia, Africa, the Americas and Australia (Zahid et al., 2008). The pest causes a yield loss of 2–84% in mungbean amounting the US $30 million (Zahid et al., 2008). The larvae damage all the stages of the crop including flowers, stems, peduncles, and pods; however, heavy damage occurs at the flowering stage where the larvae form webs combining flowers and leaves (Sharma et al., 1999). Cowpea aphid, *Aphis craccivora* Koch., sucks plant sap that causes loss of plant vigour and may lead to yellowing, stunting or distortion of plant parts. Further, aphids secrete honeydew (unused sap) that leads to the development of sooty mould on plant parts. Cowpea aphid also acts as a vector of bean common mosaic virus. Bruchids are the most important stored pests of legume seeds worldwide. They infest seeds both in field and in the storage, however, major damage is caused in storage. Bruchid damage can cause up to 100% losses within 3–6 months, if not controlled (Tomooka et al., 1992; Somta et al., 2007). Twenty species of bruchids have been reported infesting different pulse crops (Southgate, 1979). Of these, the Azuki bean weevil (*Callosobruchus chinensis* L.) and cowpea weevil (*Callosobruchus maculatus* Fab.) are the most serious pests of mungbean. The cryptic behaviour of bruchids where the grubs feed inside the legume seeds makes it easy to spread them through international trade.

## Breeding for Insect Resistance

Identification of sources of resistance is important for the introgression of resistance into cultivars through breeding. The primary gene pool forms the first choice for the breeder for source of resistance. The secondary and tertiary gene pools provide further choices of variation to be incorporated into the crop. Although a number of screening methods have been developed, lack of uniform insect infestation across seasons and locations in some key pests, whose rearing and multiplication is difficult on artificial diets, is highly challenging for screening plants against insect-pests. For pod borers, screening in field, and greenhouse conditions is generally done by releasing ten first-instar larvae on the plant placed in net wire framed cage (40 cm in diameter, 45 cm long) under no-choice and free choice conditions (Sharma et al., 2005). Under laboratory conditions, the easiest and the most reliable technique used for screening plants for pod borer and foliage feeding insects is detached leaf bioassay techniques (Sharma et al., 2005). This technique is very useful to screen the germplasm where antibiosis and non-preference are important components of plant resistance. Under field conditions, screening is also done by augmenting insect populations, planting date adjustment, tagging the inflorescences and plant grouping according to maturity and height (Sharma et al., 2005). For screening against *Maruca*, plant phenology is an important criterion to be taken into consideration (Dabrowski et al., 1983; Sharma et al., 1999). Plants are screened for resistance on the basis of the number of shoots prior to flowering and the number of eggs per plant during the early stages of the crop (Oghiakhe et al., 1992). Whitefly, thrips, and cowpea aphid resistance screening in mungbean is done on the basis of the number of insects and scoring the plants for insect damage on a visual rating scale (Taggar and Gill, 2012). Screening for bruchid resistance is done by using small plastic cups with 10–50 seeds in a no-choice or free-choice conditions and releasing up to five pairs of newly emerging adults (Somta et al., 2007, Somta et al., 2008).

To breed for resistance to insect-pests, understanding plant-insect interactions is very important. Some of the important parameters for successful breeding for insect resistance is to understand the biology of the insect pest, infesting stage and the biochemical and molecular aspect of insect-plant interactions. The role of various agro-ecological and environmental conditions along with uniform insect infestation is very important as the evaluation techniques, insect population and plant ecology depend on these factors. Further, it is important to have an optimum population build-up of the insect-pests during the most vulnerable stage of the crop. Uniform infestation at appropriate stages of plant development plays an important role in identifying insect-resistant genotypes and to reduce or eliminate the escapes (Maxwell and Jennings, 1980). Basic strategies in breeding for insect resistance are to identify the resistance coding genes from wild/cultivated species and introgress them into improved lines through recombination, hybridization, and selection. Though conventional plant breeding has some limitations it has contributed to significant improvement in yield and disease and insect resistance in mungbean (Fernandez and Shanmugasundaram, 1988). Induced mutation by using physical and chemical mutagens have been implicated in the development of insect and disease resistant varieties along with the other target traits in mungbean (Lamseejan et al., 1987; Wongpiyasatid et al., 2000; Watanasit et al., 2001). Some of the techniques in conventional breeding to develop insect resistant cultivars include mass selection, pure line selection and recurrent selection (Dhillon and Wehner, 1991; Burton and Widstorm, 2001). Techniques such as backcross breeding, pedigree breeding and bulk selection are being used for developing insect resistance in mungbean along with improved agronomic traits.

## Sources of Resistance Against Insect-Pests

Host plant resistance plays an important role in crop protection against insect pests. The identification of new insect resistance sources provides breeders with avenues to breed for resistance to insect pests. The variability primary gene-pool available with the breeders could serve an important source for various traits including insect resistance. Generally, many valuable genes that confer resistance to insect pests can be found in the wild species and/or non-domesticated crop relatives (Sharma et al., 2005). Extensive screening studies have been carried out under controlled and natural conditions to identify insect resistance sources in mungbean (**Table 3**). For stem fly, very few studies have been carried out for the identification of resistant sources in mungbean. World Vegetable Center and The International Center for Tropical Agriculture (CIAT) identified some stem fly resistant genotypes, which have been used as potential sources in breeding for resistance against stem fly (Talekar, 1990; Abate et al., 1995). CIAT identified G 05253, G 05776, G 02005, and G 02472 as highly resistant to stem fly. Co 3 has been reported as resistant to *Ophiomyia centrosematis* (De Meijere) (Devasthali and Joshi, 1994). Some of the whitefly resistant sources have been identified globally and used to breed for resistance to this pest. Abdullah-Al-Rahad et al. (2018) reported Bari Mung -6 as resistant to whitefly and cowpea aphid under natural infestation. Sources of resistance to both seedling and flower thrips have been identified in mungbean under natural and artificial infestation in mungbean (**Table 3**). Breeding for resistance to spotted pod borer has lead to the identification of some of the sources of resistance in mungbean (Chhabra et al., 1988; Sahoo et al., 1989; Gangwar and Ahmed, 1991; Sahoo and Hota, 1991; Bhople et al., 2017). In mungbean, not much work has been done to identify the sources of resistance against cowpea aphid. Just a couple of resistant sources are available (Bhople et al., 2017; Abdullah-Al-Rahad et al., 2018).

**Table 3:** Resistant sources of mungbean against insect pests.

Insect pest	Genotype(s)	Resistance level*	Country	References
Stem fly (*Ophiomyia* spp.)	V2396, V3495, V4281	R	Taiwan	Talekar (1990)
	G05253, G05776, G02005, G02472	R	Africa	Abate et al. (1995)
	Co 3	R	India	Devasthali and Joshi (1994)
	Chai Nat 72 (CN72)	MR	Thailand	Watanasit et al. (2001)
	V3726	R	Myanmar	Thi et al. (2005)
	BM 4 and Vaibhav	R	India	Bhople et al. (2017)
Whitefly (*Bemisia tabaci*)	ML 1, ML 6, ML 7, P 290, P 292, P 131, P 293, P 325, P 364, 11,148	MR	India	Kooner et al. (1997)
	ML 1265, ML 1229	R	India	Kooner and Cheema (2007)
	NM 92, NM 98	MR	Pakistan	Khattak et al. (2004)
	99.CMG-059, NM 2003-06, NM. 2003-24, NM. 2003-26, NCM. 258, PDM-54	MR	Pakistan	Shad et al. (2006)
	VBN 2, CO 8, VGG10-002	MR	India	Sekar and Nalini (2017)
	KM 200	MR	India	Panduranga et al. (2011)
	NM 04-2-38, NM 10-12-1, NM 46-5-2-21, NM 013, NM 0183, NM 04-1-11, NM 15-11	MR	Pakistan	Akhtar et al. (2011)
	MH 3153, NM-92, NM-2006, Azri 2006, NM-121	MR	Pakistan	Nadeem et al. (2014), Muhammad et al. (2018)
	TMB-36, RMG-1004	R	India	Singh and Singh (2014)
	PKV Green Gold	R	India	Bhople et al. (2017)
	Bari Mung-6	R	Bangladesh	Abdullah-Al-Rahad et al. (2018)
	MDGVV-16	R	India	Chauhan et al. (2018)
	CO 3, CO 4, CO 5	MR	India	Lal (1987)
Thrips (*Megalurothrips* spp., *Thrips palmi*)	SML 77, UPM 82-4, Pusa 107	R	India	Malik (1990)
	NM-92	R	Pakistan	Khattak et al. (2004)
	MGG 362, MGG 365	MR	India	Sandhya Rani et al. (2008)
Spotted pod borer (*Maruca* spp.)	LU-3, LU-15, LU-33, LU-173, LU-190, LU-196, LU-397, LU-426, LU-434	MR	India	Chhabra et al. (1988)
	J-1, LM-11, P-527, P-536	MR	India	Lal (1987)
	ML-65, B-101, B-103	MR	India	Gangwar and Ahmed (1991)
	PKV Green Gold	R	India	Bhople et al. (2017)
	KM-9-128, KM-9-136, RMG-492, LGG-527, LGG-538, MGG-336, KM-8-655, and MGG-335	MR	India	Sandhya Rani et al. (2014, Sandhya Rani et al., 2015)
	PDM-54-146, ML 131, ML 372	R	India	Sahoo et al. (1989)
	JRUM1, JRUM11, JRUM33, DP1703, LAM 14-2, UPM-83-6, UPM 83-10	R	India	Sahoo and Hota (1991)
	RVSm-11-9	MR	India	Singh and Singh (2014)
	LGG 505, ML 267, LGG 502, LGG 407, LGG 460, LGG 485	R	India	Swarnalatha (2007).
	CGG 08-007, CGG 08-028, ML 337, ML 5, MH 85-61, ML 325	R	India	Soundararajan et al. (2010)
	PM 10-18	R	India	Kumar and Singh (2017)
Cowpea aphid (*Aphis craccivora*)	Bari Mung-6	R	Bangladesh	Abdullah-Al-Rahad et al. (2018)
	Phule M702-1	R	India	Bhople et al. (2017)
Bruchid (*Callosobruchus* spp.)	V2709, V2802, V1128, V2817	R	Thailand	Somta et al. (2008)
	TC1966	R		Tomooka et al. (1992), Watanasit and Pichitporn (1996)
	TC1966	R		Fujii and Miyazaki, 1987; Kitamura et al., 1988; Fujii et al., 1989
	V2709, V2802	R	Taiwan	Talekar and Lin (1981, Talekar and Lin, 1992), AVRDC (1991)
	Zhonglv 3, Zhonglv 4, Zhonglv 6	R	China	Yao et al. (2015)
	Jangan	R	Korea	Hong et al. (2015)
	VC1535-11-1-B-1-3-B, VC2764-B-7-2-B, VC2764-B-7-1-B, VC1209-3-B-1-2-B, VC1482-C-12-2-B	R	Taiwan	AVRDC (1988)

*R, Resistant; MR, Moderately resistant.

Despite screening a large number of lines against bruchids, only a few resistant sources have been identified till date. These include V2709, V2802, V1128, and V2817 (Somta et al., 2008). The first bruchid resistant source was TC1966, a wild mungbean (*V. radiata* var. *sublobata* (Roxb.) Verdc.), collected in Madagascar and was used as a source of resistance (Tomooka et al., 1992; Watanasit and Pichitporn, 1996). TC1966 showed complete resistance to *C. maculatus* and *C. chinensis* and the resistant reaction was observed to be controlled by a single dominant gene, *Br* (Fujii and Miyazaki, 1987; Kitamura et al., 1988; Fujii et al., 1989). However, they found linkage drag that resulted in pod shattering in the cultivars developed using TC 1966 (Watanasit and Pichitporn, 1996). Two mungbean lines, V2709 and V2802 were identified by the World Vegetable Center with complete resistance to bruchids and have been extensively used in breeding programs to develop bruchid resistant mungbean (Talekar and Lin, 1981; AVRDC, 1991; Talekar and Lin, 1992). V2709 has been used as a source of resistance to develop three bruchid-resistant lines (Zhonglv 3, Zhonglv 4, and Zhonglv 6) in China (Yao et al., 2015) and, one bruchid-resistant variety (Jangan) in Korea (Hong et al., 2015). Somta et al. (2008) identified two mungbean cultivated lines, V1128 and V2817 as resistant to *C. maculatus*. At the World Vegetable Center, bruchid resistance from two black gram accessions, VM2011 and VM2164 was introgressed into mungbean successfully (AVRDC, 1987). Out of 101 breeding lines screened against bruchids, five lines (VC1535-11-1-B-1-3-B, VC2764-B-7-2-B, VC2764-B-7-1-B, VC1209-3-B-1-2-B, and VC1482-C-12-2-B) were reported as tolerant to bruchids (AVRDC, 1988). Recently, World Vegetable Center has developed promising lines that are resistant to bruchids, thrips and cowpea aphid (ACIAR, 2018; ACIAR, 2019).

Among insect-pests, bruchid resistance in mungbean has been extensively studied using the molecular techniques. However, QTL mapping for resistance to field insect-pests that are common in legumes has been studied common bean and cowpea. In common bean, *Empoasca* spp. (Murray et al., 2004), *T. palmi* (Frei et al., 2005), *Apion godmani* Wagner (Blair et al., 2006) and bruchids (Blair et al., 2010), while in cowpea, *Megalurothrips sjostedti* (Trybon) (Omo-Ikerodah et al., 2008) and *A. craccivora* (Huynh et al., 2015) have been studied in detail. The stem fly resistance in mungbean has been found to be governed by additive, dominance and epistasis mechanisms (Distabanjong and Srinives, 1985). The wild species of mungbean TC 1966, which is resistant to *C. maculatus, C. chinensis, C. analis* and *C. phaseoli* has been widely used by breeders to develop bruchid resistant lines by crossing with agronomically superior cultivars (Fujii et al., 1989; Talekar and Lin, 1992; Tomooka et al., 1992; Somta et al., 2007). Molecular techniques have been utilized to identify bruchid resistant mungbean, locate genes that code for bruchid resistance, clone them genes and develop molecular markers for mapping bruchid resistance (Tomooka et al., 1992; Tomooka et al., 2000; Somta et al., 2008; Schafleitner et al., 2016). The selection efficiency and reduction in tests for screening of breeding material against insect pests including bruchids has been increased by the molecular markers developed (Schafleitner et al., 2016).

Various molecular markers such as restriction fragment length polymorphism (RFLP), RAPD, single nucleotide polymorphism (SNP) and SSR have been used to map bruchid resistance in mungbean (Young et al., 1992; Villareal et al., 1998; Chen et al., 2007; Chotechung et al., 2011), most of them are qualitative and the results are based on phenotypic data. In TC1966, bruchid resistance has been mapped using RFLP (Young et al., 1992). They mapped 14 linkage groups containing 153 RFLP markers of 1,295 centiMorgans (cM) with an average distance of 9.3 cM between the markers. The analysis of 58 F_2_ progenies from a cross between TC1966 and a susceptible mungbean cultivar showed that an individual F_2_ population possess a bruchid resistance gene within a tightly linked double crossover and was used for the development of bruchid resistant mungbean. A population derived from a cross between the cultivar Berken and ACC41 (a wild mungbean genotype, *V. radiata* subsp. *sublobata*) using RFLP probes were used to develop a linkage map (Humphry et al., 2002). The mungbean bacterial artificial chromosome libraries have been developed by *STSbr1* and *STSbr2* [polymerase chain reaction-based markers] (Miyagi et al., 2004). The authors reported close linkage in a recombinant inbred line (RIL) population between ACC41 and ‘Berken’. Further, Sarkar et al. (2011) showed that *STSbr1* amplified a 225bp fragment in *V. sublobata* accession (sub2) and 12 other cultivars that were resistant to bruchids. Though RAPD markers are fast and simple, the distance between them is high from the bruchids resistant gene. RAPD markers for bruchid resistance have also been used with a mapping population from RIL and near-isogenic line (NIL; B4P 5-3-10, B4P3-3-23, DHK 2-18, and B4Gr3-1 with bruchid resistant genes from Pagasa 5, Pagasa 3, VC 1973A and Taiwan Green, respectively by using TC 1966 as a resistance source (Villareal et al., 1998). NILs were differentiated by using 31 RAPD markers from which 25 showed co-segregation in the RIL population. A RIL population obtained from crossing ‘Berken’ (bruchid-susceptible line) with ACC41 (bruchid-resistant line) was used to map the Br1 locus (Wang et al., 2016). Ten RAPD markers were identified by Chen et al. (2007) for bruchid resistance in 200 RILs from a cross between TC1966 and NM 92. These included UBC66, UBC168, UBC223, UBC313, UBC353, OPM04, OPU11, OPV02, OPW02, and OPW13. Out of these, four markers (OPW02, UBC223, OPU11, and OPV02) were closely linked. For bruchid resistance in mungbean, a few SSR markers have been reported. These include SSRbr1, DMB-SSR158, and GBssr-MB87 (Miyagi et al., 2004; Chotechung et al., 2011; Chen et al., 2013; Hong et al., 2015). In V2802 and TC 1966, chromosome 5 possess the DMB-SSR 158 marker associated with *Vradi05g03940-VrPGIP1* and *Vradi05g03950-VrPGIP2* genes, which code for polygalacturonase inhibitor involved in bruchid resistance (Chen et al., 2013; Chotechung et al., 2016). The major QTL in TC1966 and DMB-SSr 158 marker are <0.1cM away from the bruchid resistant gene (Chen et al., 2013). Also, QTL *qBr* has been reported between markers VrBr-SSR013 and DMB-SSR158 at the same position.

The sequence-changed protein genes (SCPs) and differentially expressed genes (DEGs) retain the transcript diversity and specificity of the *Br* genes (Liu et al., 2016) and the variations in DEGs promoter and of SCPs can be potential markers in breeding for resistance against bruchids. Two QTLs, *MB87* and *SOPU11* have been reported to be associated with bruchid resistant genes in the study from a population developed from crossing Sunhwa (susceptible) and Jangan (resistant variety developed from back crossing with V2709) (Hong et al., 2015). Mei et al. (2009) reported a QTL in wild mungbean ACC41 that accounts for about 98.5% of bruchid resistance.

Recently, SNP markers have gained high momentum for use in breeding for pest and disease resistant plants. Their abundant, ubiquitous nature in the genome and readily availability for genotyping makes them very useful (Brumfield et al., 2003). Further, being co-dominant, single-locus, and biallelic markers, the SNPs are unique for use in breeding programs. Owing to the small genome size of mungbean (515 Mb/1C), the full genome sequencing or a reduced representation library sequencing are possible that would lead to the generation of many SNP markers (Moe et al., 2011). Further, SNPs have been extensively studied in breeding for resistance in mungbean against stink bug, *Riptortus clavatus* and adzuki bean weevil, *C. chinensis* (Moe et al., 2011; Schafleitner et al., 2016). Schafleitner et al. (2016) identified dCAPS2, dCAPS3, CAPS1, and CAPS12 SNP markers for bruchid resistance in mungbean. Despite being physically mapped to different chromosomes, these markers showed genetic linkage by co-segregation at the proportions of 96.5% in the F_3_ families of the crosses TC 1966 X NM 92 and V2802 X NM 94. They reported that in both crosses, the QTL for the bruchid resistance was mapped to chromosome 5 and the markers showed the prediction of 100%. Kaewwongwal et al. (2017) reported that *VrPGIP1* and *VrPGIP2*, which are tightly linked genes confer bruchid resistance in V2709. They identified two alleles for VrPGIP1 and VrPGIP2 in V2709 as *VrPGIP1-1* and *VrPGIP2-2*, respectively.

The next generation sequencing (NGS) technologies are being utilized to develop SNPs used for genotyping several traits and increase the amounts of transcripts much higher than the cloning and Sanger sequencing approaches in plants and animals. The genetic complexities of various traits including resistance to biotic and abiotic stresses are being studied using genotyping by sequencing (GBS) methods. Some of the areas in which GBS has been utilized include purity testing, genetic mapping, MAS, marker-trait associations, and genomic selection (Schafleitner et al., 2016). Schafleitner et al. (2016) used GBS technology on populations derived from TC1966 (wild mungbean accession-bruchid resistant) and V2802 (a cultivated mungbean accession) with bruchid susceptible lines, NM 92 and NM 94. A total of 32,856 SNPs were obtained, out of which 9,282 SNPs were scored in RIL populations. Finally, 7,460 SNP sequences were aligned to 11 chromosomes and 1,822 were aligned to scaffold sequences. It has been reported that SuperSAGE in combination with the NGS has been applied to study the biotic and abiotic stress resistance/tolerance in some legumes (Rodrigues et al., 2012; Almeida et al., 2014), however, such combinations have not been studied in detail for insect resistance. RNAseq technique is very important to study the pest and disease resistance in plants in a given situation. In RNAseq, sequencing of all the transcripts that are expressed in response to pest pressure is developed and is highly powerful as the transcriptomes are synthesised *de novo* and can also be used to compare the expression of genes in different insect pressures. Additionally, RNAseq can be used to study the simultaneous expression of genes both in plant and in the pest in a given situation (Liu et al., 2012). Genome-wide transcriptome profiling techniques provide the expression of a huge number of genes in response to insect damage, however, it is challenging to identify which of them are involved in resistant plant phenotypes. The studies on the co-localization of these genes with QTLs and functional genomics has been quite helpful, however, it will be critical to study the generation and application of high-throughput reverse genetic platforms. Though functional genomics is applied to understand the genetic basis of resistance and is implicated in breeding for resistance against insect-pests, further in-depth investigations are needed to stabilize the insect resistance in mungbean. Furthermore, identification of molecular markers linked to genes/QTLs controlling insect-pest resistance has been studied in many legumes, only in a few cases, these markers have been used in MAS breeding, the main constraint being the large distance between the markers and the gene/QTL controlling resistance (Shi et al., 2009; Schafleitner et al., 2016).

## Abiotic Stresses in Mungbean

**Figure 1:**
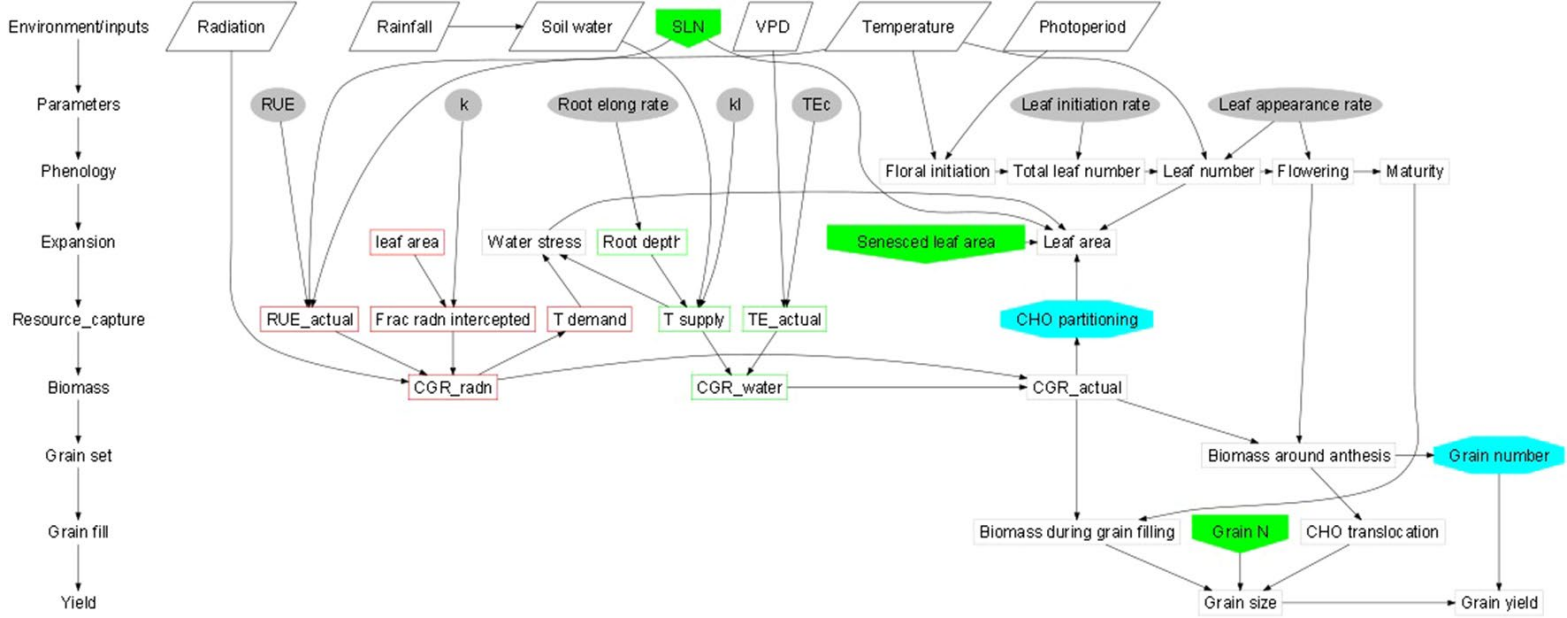
Schematic representations of crop growth and development dynamics (Generic template; Connections between the two schematics are shown by the shaded boxes); [Hammer et al., 2010: https://doi.org/10.1093/jxb/erq095].

**Figure 2:**
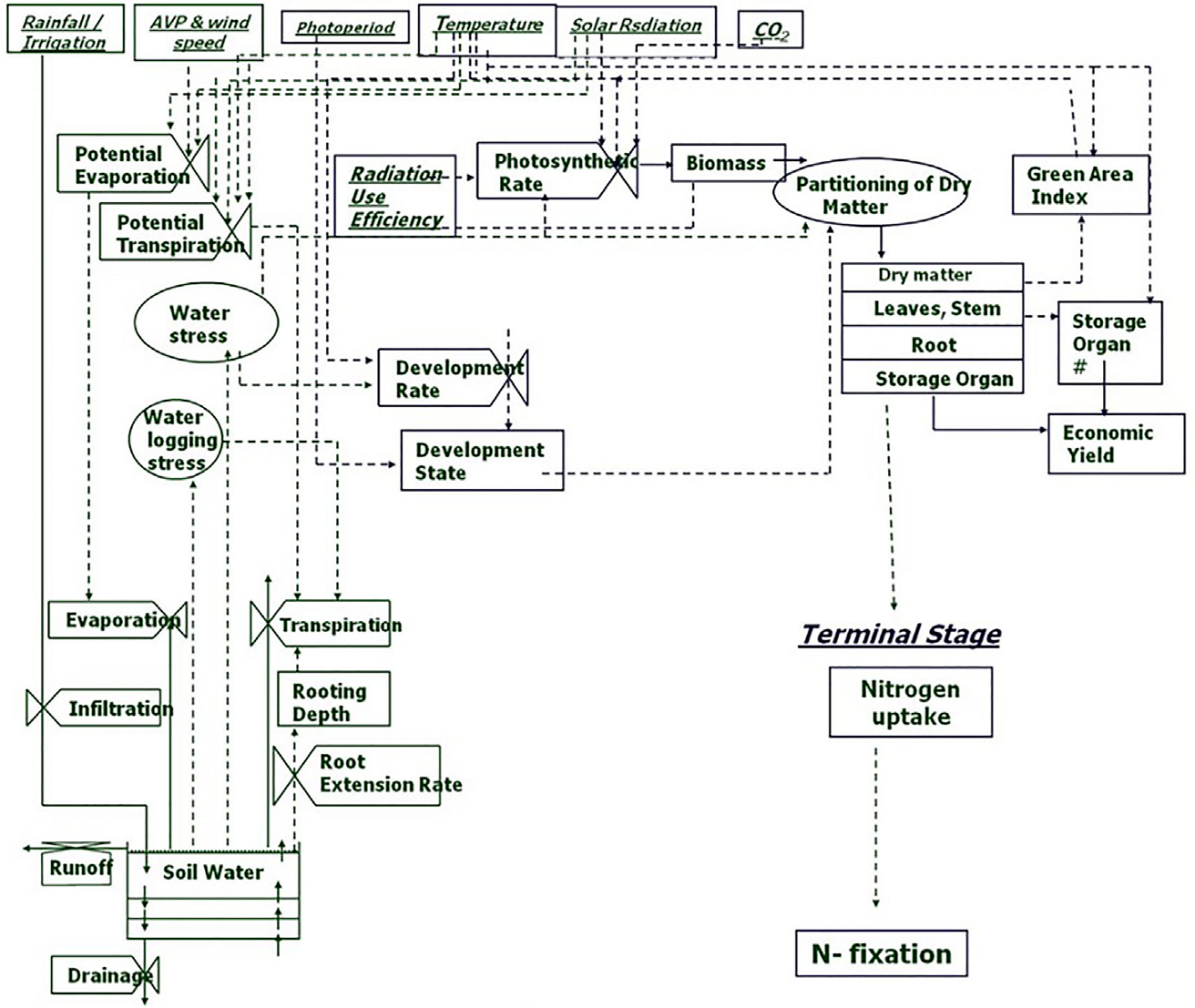
Process chart of mungbean growth model (MungGro) [Biswas et al., 2018]

Abiotic stresses negatively influence plant growth and productivity and are the primary cause of extensive agricultural losses worldwide (Arun and Venkateswarlu, 2011; Ye et al., 2017). Reduction in crop yield due to environment variations has increased steadily over the decades (Boyer et al., 2013). Abiotic stresses include extreme events and factors related to atmosphere (heat, cold, and frost); water (drought and flooding); radiation (UV and ionizing radiation); soil (salinity, mineral or nutrient deficiency, heavy metal pollutants, pesticide residue, etc.) and mechanical factors (wind, soil compaction) (HanumanthaRao et al., 2016). Crops utilize resources (light, water, carbon and mineral nutrients) from their immediate environment for their growth. The microenvironment and the management practice of cultivation influence crop growth and development directly (**Figure 1**). Climate change further adds to the complexity of plant-environment interactions (Goyary, 2009). The eco-physiological models that integrate the understanding of crop physiology and crop responses to environmental cues from detailed phenotyping are therefore used to understand the impact of environmental factors on crop growth and development, predict yield/plant response and also assist in developing management strategies (**Figure 2**) (APSIM: Chauhan et al., 2010; MungGro: Biswas et al., 2018). The plant response to abiotic stress at the cellular level is often interconnected (Beck et al., 2007) leading to molecular, biochemical, physiological and morphological changes that affect plant growth, development and productivity (Ahmad and Prasad, 2012). Several crop production models project a reduction in the crop yields of major agricultural crops mostly due to climate change (Rosenzweig et al., 2014), which tend to make crop growth environment unfavorable due to abiotic stresses. Such efforts in crops like mungbean is rare and requires a special attention. In the current era, environmental stresses are a menace to global agriculture and there is a need to emphasize trait based breeding to ensure yield stability across the locations as well as crop seasons. Efforts are underway to develop new tools for understanding possible mechanisms related to stress tolerance and identification of stress tolerance traits for promoting sustainable agriculture (Cramer et al., 2011; Fiorani and Schurr, 2013). Basic tolerance mechanisms involve the activation of different stress-regulated genes through integrated cellular as well as molecular responses (Latif et al., 2016). Plants respond to their immediate surroundings in diverse ways, which assist the cells to adapt and achieve cellular homeostasis manifested in phenotypes of plants under particular environment (James et al., 2011). While breeding lines are regularly phenotyped for easily visible traits including growth and yield components, many traits that contribute to stress tolerance are ignored. This can be largely due to feasibility of measuring these traits precisely and rapidly. Hence, recent phenotyping tools deploy image capture and automation in advanced plant phenotyping platforms. These recent efforts are expected to boost efforts to translate basic physiology of crop plants into products with practical values to support breeding program in harsh environments (viz., stresses like salinity, soil moisture, extreme temperatures etc) explained in the following section.

## Salinity

In agriculture, soil salinity has been a threat in some parts of the world for over 3000 years (Flowers, 2006) and it has been aggravated by irrigation water sourced through surface irrigation in arid and semi-arid environments (HanumanthaRao et al., 2016). Salt stress mainly in most of the crops reduces seed germination, fresh and dry biomass, shoot and root length, and yield attributes of mungbean (Promila and Kumar, 2000; Rabie, 2005; Ahmed, 2009). It affects root growth and elongation, thereby, hampering nutrient uptake and distribution. Root growth was significantly reduced with higher Sodium Chloride (NaCl) (NaCl) concentrations. Nevertheless, BARI Mung4 showed better performances at higher NaCl concentration considering a yield-contributing character. Nodules/plant decreased with the increase of salinity although the nodule size increased (Naher and Alam, 2010). Being polygenic in nature, salinity tolerance is genotype-dependent and growth stage-specific phenomenon, therefore, tolerance at an initial (seedling) stage may not be corroborated with tolerance at later growth (maturity) stages (Sehrawat et al., 2013). It also involves multidimensional responses at several organ levels in plants (e.g., tissue, molecular, physiological and plant canopy levels) (HanumanthaRao et al., 2016). Because of this complexity and lack of appropriate techniques for introgression, little progress has been achieved in developing salt-tolerant mungbean varieties over years (Ambede et al., 2012; HanumanthaRao et al., 2016). Appreciable improvement in salt tolerance of important crops (barley, rice, pearl millet, maize, sorghum, alfalfa, and many grass species) have been attained in the past, but not in legumes in general and mungbean in particular (Ambede et al., 2012). Rapid screening methods are required to identify putative donor parents in a breeding program (Saha et al., 2010). In a comprehensive study, Manasa et al. (2017) screened 40 mungbean lines sourced from World Vegetable Center for salinity tolerance using Salinity Induction Response (SIR) technique at the seedling as well as at whole plant levels by canopy phenotyping assay under 150 and 300 mM NaCl stress scenario. The results showed a marked reduction in growth and yield performances of both tolerant and susceptible lines, but a few lines displayed a relatively better biomass and pod yield on par with non-stressed control plants. The intrinsic ability of salt portioning to vacuole (more influx of Na^+^ ions) by tolerant lines during high salt concentration in the cytocol could be one of the reasons for their tolerance. Based on the extent of salt tolerance both at seedling and whole plant stages, a few salt tolerant (EC 693357, 58, 66, 71, and ML1299) lines were identified (Manasa et al., 2017) for further validation under field conditions.

## Soil Moisture Stress

The response of legumes to the onset of drought vary and the final harvestable yield will significantly be reduced (Nadeem et al., 2019). Global climate change attributes erratic prediction in drought episodes and its control of crop yields. Being grown on marginal lands, mungbean is largely considered as a drought tolerant (grow with a limited soil moisture). However, like any other plants, it responds to a decrease in available soil moisture by reducing its growth and hence productivity. It is evident from the experiment that 30% decrease in water supply relative to water optimum for crop growth results in nearly 20% decrease in seed weight per plant if the soil moisture stress imposed around a vegetative stage. The plants subjected to stress during flowering showed 50 to 60% decrease in seed yield (Fathy et al., 2018). Soil moisture stress did not affect the number of pods per plant as severely as it did for seed weight or biomass per plant in this experiment, clearly indicating that seed formation or filling is the most sensitive to soil moisture stress. It is also suggested that the dry matter partitioning is one of the potential screening trait for drought tolerance in mungbean (Hossain et al., 2010; Nadeem et al., 2019). When the drought stress was severe enough to reduce plant biomass per m^2^ from 359 to 138 g, the resultant reduction in pod number was nearly 50% and the same for seed yield was nearly 60% relative to well-watered plants (Kumar and Sharma, 2009).

The decrease in total plant dry weight and harvest index were the main reasons for reduced seed yield due to drought stress in mungbean (Sadasivan et al., 1988; Thomas et al., 2004). Significant reduction in pod initiation and pod growth rates were the major responses to soil moisture stress during flowering and pod-filling stages (Begg, 1980). Water stress during flowering results in reduced yield mainly due to flower abscission (Moradi et al., 2009). The relative water content in leaves and partitioning of biomass have been sighted as the traits contributing to tolerance to drought in summer mungbean (Kumar and Sharma, 2009). Yield loss of 31-57% at flowering and 26% at post flowering/podding stages in mungbean due to drought stress was reported by Nadeem et al. (2019). The drought-induced imbalance in electrons produced and consumed during the photosynthetic process gives rise to harmful superoxide molecules, which have been cited as a major reason for damages at the cellular level. Hence, key factors that can alleviate oxidative stress are the focus of research for alleviating drought stress. Recent studies infer that alleviation of drought-caused oxidative stress depends largely on the status of Ascorbic acid and Glutathione pools in reduced and oxidative stages (Anjum et al., 2015). There is a need to explore genetic variation for these traits and possibility of introgressing the relevant genes for improving drought tolerance in mungbean. Decreased leaf water potential was associated with reduced activity of nitrogenase, glutamine synthetase, asparagine synthetase, aspartate aminotransferase, xanthine dehydrogenase and uricase that are associated with nitrogen fixation (Kaur et al., 1985). New insights into these metabolites and enzymes can be obtained to understand their roles through recently evolved metabolomics.

Water stress-induced inhibition of hypocotyl elongation is more conspicuous in separated cotyledons than the intact ones. It is necessary to check if the larger cotyledons can be the solution for better plant establishment under soil moisture stress. When two mungbean genotypes exhibiting more than two-fold variation in leaf water loss were explored for the genetic variation in their physiological and molecular responses to drought, efficient stomatal regulation was observed in water saving low leaf water loss (LWL) genotype (Raina et al., 2016). The stomatal closure under drought was accompanied with a concomitant down-regulation of farnesyl transferase gene in this genotype. However, other genotypes had a cooler canopy temperature facilitated by a branched root system that allowed better extraction of soil moisture (Raina et al., 2016). These mechanisms and traits of mungbean are suitable for harsh environments but needs a prioritization based on the type of drought and agro-ecological features. The other important key physiological traits viz., water use efficiency, root growth/biomass, carbon isotope discrimination (∆^13^C) and leaf temperature (Canopy temperature difference), may be beneficial for screening mungbean for drought tolerance.

## High Temperature or Heat Stress and Increasing Atmospheric Carbon Dioxide (CO_2_)

Of the various environmental stresses that a plant can experience, temperature has the widest and far-reaching effects on legumes. Temperature extremes, both high (heat stress) and low (cold stress), are injurious to plants at all stages of development, resulting in severe loss of productivity. Legumes, such as chickpea, lentil, mungbean, soybean, and peas, show varying degrees of sensitivity to high and low-temperature stresses, which reduces their potential performance at different developmental stages such as germination, seedling emergence, vegetative phase, flowering, and pod/seed filling phase (HanumanthaRao et al., 2016; Sharma et al., 2016). The optimum temperature for growth and development of mungbean is 28–30°C and the range under which plant continues to develop seed is 33–35°C. Each degree rise in temperatures above optimum reduces the seed yield by 35–40% relative to the plants grown under optimum temperature (Sharma et al., 2016).

Temperatures >45°C that often coincides at flowering stage can lead to flower abortion and yield losses. Sharma et al. (2016) evaluated the effect of high temperature on different mungbean lines for vegetative and reproductive performances using Temperature Induction Response (TIR) and physiological screening, techniques at seedling and whole plant levels. The promising tolerant lines were shortlisted for further investigation at the whole plant level. These lines were grown in containers under full irrigation in outdoors; screened for growth and yield traits at two sowings: normal sowing (NS), where day/night temperatures during reproductive stage were <40/28°C, and late sowing (LS), where temperatures were higher (> 40/28°C). The leaves of LS plants showed symptoms of leaf rolling and chlorosis and accelerated phenology lead to sizable marked reduction in leaf area, biomass, flowers and pods. Interestingly, shortening of flowering and podding duration was also observed.

To address ever-fluctuating temperature extremes that various legumes get exposed to, efforts are being made to develop heat-tolerant varieties through conventional breeding methods (exposing breeding lines to open air growing seasons having high temperature episodes either throughout the growth stages or specific to flowering or reproductive phase) in order to select promising tolerant lines. Subsequently subject these shortlisted entries to varied growing environments that coincide with drier/heat periods for confirmatory validation to identify true-genotypes to engage them in heat stress breeding programs. With the advancement of `omics’ era, phenomics platform (phenotyping) can conveniently be applied to screen field shortlisted or promising sub-set of candidates with more precisely conditioned high-temperature regimes (at customized growth periods) to identify true types along with expressed plant architectures. Tolerance to suboptimal temperatures has not been studied extensively in crops like mungbean. However, for the improvement in grain yield of this crop in hilly areas or in higher latitudes it is necessary to introgress traits associated with cold or low-temperature tolerance.

Increasing atmospheric CO_2_ concentration along with temperature also pose a constraint to plant growth and development, which would be more pronounced in C_3_ plant species (like mungbean) than C_4_. Some of the physiological functions (activation of carboxylating enzymes, photosynthetic rates, cell expansion, carbohydrate synthesis etc) will be enhanced which have an impact on leaf area and biomass associated improvements. An improved biomass by virtue of increased leaf expansion may not always result in higher yield levels. However, in mungbean, higher pod and seed yields were documented when a few high temperature tolerant genotypes exposed to elevated CO_2_ of 550 ppm compared to ambient CO_2_ of 400 ppm (Bindumadhava et al., 2018). However, molecular mechanism governing aggravated metabolic functions at different growth stages is still unclear and possibility of employing CO_2_ fertigation as a breedable trait needs more research attention in days to come from the context of changing global climate.

## Waterlogging

Anthropogenic studies reveal that the frequency and severity of flooding events increase with climate change (Arnell and Liu, 2001). Waterlogging adversely affects germination, seedling emergence and growth, crop establishment and root and shoot growth (Bailey-Serres and Voesenek, 2008; Toker and Mutlu, 2011). Heavy rains during pod ripening stage results in premature sprouting, leading to inferior seeds. Mungbean is predominantly cultivated in rice-fallow systems and is sensitive to waterlogging (Singh and Singh, 2011). Excess rainfall in such cultivation systems can result in waterlogging wherein roots are completely immersed in water and shoots (sometimes) are partially or fully submerged. Ahmed et al. (2013) highlighted the biochemical mechanisms *viz*., increased availability of soluble sugar, enhanced enzymatic activity of glycolytic pathway antioxidant defense mechanism, and altered aerenchyma formation help plants withstand waterlogging. In addition to the deficiency of oxygen, waterlogging can alter the mineral nutrient composition accessible for plants and needs to be considered during genetic crop improvement (Setter et al., 2009). Spring grown crops are more prone to water stress as the rainfall is scanty and farmers mostly prefer to grow this crop on residual moisture. Therefore, cultivating short duration cultivars may help in escaping terminal moisture stress (Pratap et al., 2013).

## Breeding for Abiotic Traits

At the plant level, there were several satisfying attempts in mungbean to screen and identify tolerant types for high temperature (heat stress), salinity, waterlogging, and water stress from physiological, biochemical, and molecular perspectives (Kaur et al., 2015; HanumanthaRao et al., 2016; Bhandari et al., 2017; Manasa et al., 2017; Sehgal et al., 2018). The breeding lines selected and identified for these aforementioned stresses would form a panel of donor resources for future trait-navigated crop improvement (**Table 4**).

**Table 4:** Tolerant/resistant sources of mungbean against abiotic stresses.

Abiotic stress/s	Source of tolerance	Country	Reference
Drought	K-851	India	Dutta and Bera (2008), Dutta et al. (2016)
Heat tolerance and elevated CO_2_ levels	EC693357, EC693358, EC693369, Harsha and ML1299	India	Sharma et al. (2016), Bindumadhava et al. (2018)
Drought	TCR 20	India	Tripathy et al. (2016)
Drought	SML-1411, SML-1136	India	Kaur et al. (2017)
Drought	ML 267	India	Swathi et al. (2017)
Drought	VC 2917 (seedling stage)	China	Wang et al. (2014, 2015)
Drought	V-1281, V-2013 and V-3372	Taiwan	AVRDC (1979)
Waterlogging	V 1968, V 2984, V 3092 and V 3372	Taiwan	AVRDC (1979)
Drought	VC 1163 D, VC 2570A, VC 2754 A and VC 2768 A	Taiwan	Fernandez and Shanmugasundaram (1988)
Drought & Flooding	V 1381 and VC 2778	China	He et al.(1988)
Low temperature	Perennial accessions of *V. radiata* var. *sublobata*	Taiwan	Lawn et al. (1988)
Salt	S72, H45, No. 525, Madira and RS-4	India	Maliwal and Paliwal (1982)
Salt	T-44	India	Misra and Gupta (2006)
Salt	BARI Mung-4	Bangladesh	Naher and Alam (2010)
Salt	NM 19-19	Pakistan	Shakeel and Mansoor (2012)
Salt	TCR86, PLM380, PLM562, WGG37, IC615, PLM891, IC2056, IC10492, PLM32, K851, and BB92R	India	Sehrawat et al. (2014)
Salt	EC 693357, 58, 66, 71 and ML 1299	India	Manasa et al. (2017)
Pre-harvest sprouting	Chamu 4	India	Lamichaney et al. (2017)
Heat	IPM 02-16, IPM 9901-10, IPM 409-4, IPM 02-3, PDM 139, IPM 02-1, IPM 2-14, IPM 9-43-K, PDM 288, EC 470096, IPM 2K14-9, IPM 2K14-5	India	Khattak et al. (2009)
Drought (maintaining cooler canopy traits)	VC-6173-C, IC-325770, ML 2082	India	Raina et al. (2016)

The initial phase of breeding in mungbean resulted in selecting a few locally adapted germplasm, mainly for biotic stresses resistance and high yield. While selecting for abiotic stress resistance was not practiced directly, selection for yield, plant type, and adaptation related traits indirectly lead to selection for abiotic stress resistance as well. The selection has been a useful strategy to identify superior cultivars with significant drought tolerance. Warm season food legumes generally encounter two types of drought stresses: (i) terminal drought, which is more prominent in summer/spring crops, usually coincides with late reproductive stage and increases towards generative stage, and (ii) intermittent drought, which may occur anytime during vegetative growth and results due to a break in rainfall or insufficient rains at the vegetative stage. The ranking of warm season food legumes in increasing order of drought resistance was soybean, followed by blackgram, mungbean, groundnut, bambara nut, lablab bean and cowpea (Singh et al., 1999). Fernandez and Kuo (1993) used a stress tolerance index (STI) to select genotypes with high yield and tolerance to temperature and water stresses in mungbean. Singh (1997) described the plant type of mungbean suitable for Kharif (rainy) as well as dry (spring/summer) seasons. Pratap et al. (2013) also suggested the development of short duration cultivars for Spring/Summer cultivation so that these escape terminal heat and drought stress. Cultivars with 60–65 days’ crop cycle, determinate growth habit, high harvest index, reduced photoperiod sensitivity, fast initial growth, longer pods with more than 10 seeds/pod and large seeds are more suitable to the summer season. Keeping this backdrop, a number of early maturing mungbean lines have been selected and released as commercial cultivars.

## RNAi Technology: Biotic and Abiotic Stress Resistance

**Figure 3:**
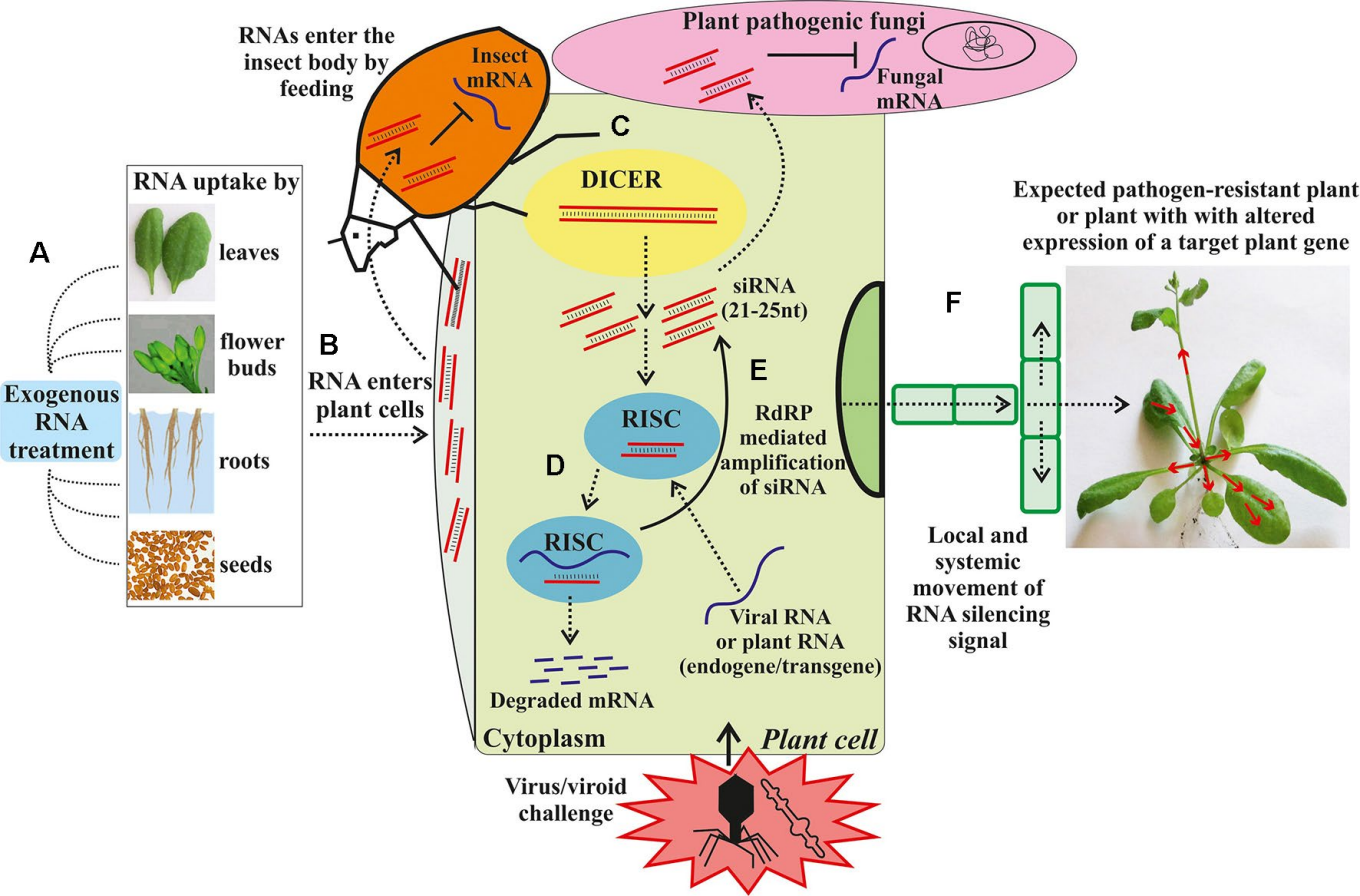
Exogenous RNA applications for RNA interference (RNAi) in plants against biotic stresses. **(A)** Exogenous artificial RNA application on the plant. **(B)** The exogenous RNAs transported into the cytoplasm. **(C)** The dsRNA or hpRNA molecules are recognized by a ribonuclease, DICER-like (DICER), which cleaves the dsRNA into siRNAs. **(D)** The siRNAs are then incorporated in the RNA-induced silencing complex (RISC) that guides sequence-specific degradation or translational repression of homologous mRNAs. **(E)** The components of the siRNA/mRNA complex can be amplified into secondary siRNAs by the action of RNA-dependent RNA-polymerase (RdRP). **(F)** Movement of the RNA silencing signal between plant cells and through the vasculature. Dashed arrows depict different steps of the RNAi induction process and dsRNA/siRNA movement between plant cells and plant pathogens. The solid arrow depicts the RdRP-mediated amplification of siRNA. Red arrows depict the local and systemic movement of the RNA silencing signal in the plant (From Dubrovina and Kiselev, 2019).

Though conventional breeding strategies have helped breeders to produce disease and insect resistant, and high yielding varieties, the challenges in the conventional breeding make it time-consuming and often leads to the transfer of undesired traits along with desired traits. Further, the functional analysis of candidate genes that code for physiological and biochemical pathways in plants responsible for resistance against diseases and insect-pests have been studied in detail in legumes. However, these studied are limited in mungbean. To further advance the functional genomic analysis of plants, gene silencing technologies using RNA interference (RNAi) or virus-induced gene silencing have been developed to study the expression or inhibition of the candidate genes (Wesley et al., 2001). RNAi technology offers a new and innovative potential tool for plant breeding for resistance/tolerance to biotic and abiotic stresses through the introduction of small non-coding RNA sequences that are able to regulate gene expression in a sequence-specific manner (**Figure 3**; Dubrovina and Kiselev, 2019). The suppression of expression of a specific gene provides an opportunity to remove or accumulate a specific trait in plants that would lead to biochemical or phenotypic changes, which in turn, provide resistance/tolerance to plants against biotic and abiotic stresses. Furthermore, RNAi-mediated gene silencing techniques can be used by plant breeders to suppress genes in full or partially using specific promoters and construct design (Senthil-Kumar and Mysore, 2010). In RNAi technology, the candidate gene activity is disrupted and or silenced in a sequence-specific manner by introducing constructs that generate double-stranded RNAs (Dennis et al., 1999). Though this technology is generally used as a pest and disease control strategy on the pest aspect, the plant-mediated or host-induced RNAi (HI-RNAi) can be used to develop the engineered crop plant material with hair-pin RNAi vector to produce dsRNA that would target the insect and pathogen genes. When the insect feeds on the plant parts, the entry of dsRNA into the insect gut will induce the RNAi activity and silence the target gene in the insect pest (Zha et al., 2011). Further, RNAi can be used to alter the gene expression in plants involved in resistance against diseases (Senthil-Kumar and Mysore, 2010) and abiotic stresses (Abhary and Rezk, 2015). Haq et al. (2010) studied the silencing of complementary-sense virus genes involved in MYMV replication in soybean by targeting a complementary-sense gene (ACI) encoding Replication Initiation Protein (Rep) against Mungbean yellow mosaic India virus. Similarly, Kumar et al. (2017) generated cowpea plants with resistance to MYMV using RNAi technology, which contained three different intron hairpin RNAi constructs. RNAi technology has been used against a number of insect-pests such as *H. armigera* by targeting the *CYP6AE14* gene 9 (Mao et al., 2007). When transcriptional factor genes of *H. armigera* were targeted by HI-RNAi, a significant reduction in mRNA and protein levels was observed that resulted in deformed larvae and larval mortality (Xiong et al., 2013). Additionally, this technology has been implicated in increasing the production of unique secondary metabolites, increasing the shelf life of the fruits, improving crop yield and improving insect and disease resistance (Abhary and Rezk, 2015). Sunkar and Zhu (2004) reported that in *Arabidopsis* plants, miRNAs are involved in tolerance against abiotic stress including cold, drought, and salinity. They further showed that exposure to higher salinity levels, dehydration, cold, and abscisic acid upregulated the expression of miR393. While RNAi technology can be used to improve biotic and abiotic stress resistance/tolerance in mungbean, large-scale field studies are needed to study any potential risks of this technology.

## Breeding Constraints for Developing Biotic/Abiotic Stress Resistant/Tolerant Mungbean

In breeding for resistance to biotic and abiotic stresses in legumes, the important factors that are taken into consideration include the genetic distance between the resistant source and the cultivars to be improved, screening methodology, inheritance pattern and the resistance traits to be improved. The genetic diversity and the genetic distances between cultivars and the resistance sources can be integrated in breeding approach such as gene pyramiding (Kelly et al., 1998; Kim et al., 2015). The important breeding approaches such as the pedigree and single seed descent methods are used to transfer the major resistant alleles and QTLs between cultivars and elite breeding lines. However, the increased genetic distances between the source and the cultivars lead to segregation of characters, which can be reduced by repeated backcrossing such as inbred-backcrossing, recurrent backcrossing, or congruity backcrossing (i.e., backcrossing alternately with either parent). During early stages of the breeding program for breeding to diseases and insect resistance, introgressing resistance alleles and QTL from wild populations, recurrent or congruity backcrossing or modifications are highly important. Although gamete selection using multiple-parent crosses (Asensio-S.-Manzanera et al., 2005, Asensio-S.-Manzanera et al., 2006) and recurrent selection (Kelly and Adams, 1987; Singh et al., 1999; Terán and Singh, 2010), respectively, could be effective, their use in the legumes where a large number of pollinations are required may not be feasible.

Linkage drag is one of the important challenges while developing the disease or insect resistant cultivars, especially when wild sources are used as donors. To reduce linkage drag, repeated backcrossings are needed (Keneni et al., 2011). Deployment of wild germplasm in resistance breeding, which is an important source of resistance introgression to commercial cultivars, is often impeded by the undesirable genetic linkages, which may result in the co-inheritance of the undesired and desired traits that may affect seed quality, germination and other traits (Edwards and Singh, 2006; Acosta-Gallegos et al., 2008; Keneni et al., 2011). Breeding for resistant to diseases and insect-pests where resistance is controlled by a single gene is easier as compared to multigenic resistance (Miyagi et al., 2004; Somta et al., 2008; War et al., 2017). The multigenic disease and insect-resistance with low dominance may result in the transfer of the undesirable traits such as leaf size, seed texture, and color along with the desired traits (Edwards and Singh, 2006). Crossing over between homologous chromosomes during meiosis is important to transfer the genes controlling desired traits and to overcome the linkage drag. For this, a large number of F_2_ populations is required to be grown to increase the recovery of new recombinants due to crossing-over.

Another very important factor impeding breeding for resistance to diseases is the development of various strains by a pathogen and to insect-pests is the biotypic variation in insect-pests. Plant genotypes that are resistant to one pathogen strain or insect biotype may be susceptible to the other strain of the same pathogen or insect biotype. Insect biotypes show genetic variability within a pest population. Biotype species are morphologically similar, however, their biological traits vary. The emergence and spread of whitefly-transmitted viruses are attributed to the evolution of virus strains, development of aggressive biotypes and increase in the whitefly population (Chiel et al., 2007). While studying the MYMV begomoviruses infecting mungbean and their interaction with *B. tabaci* in India, Nair et al. (2017) identified that a MYMV resistant NM 94 variety was susceptible to the disease in different locations. The MYMV strains identified were MYMV-Urdbean, MYMV-*Vigna* and MYMIV. They further identified that three cryptic species of *B. tabaci* are responsible for spreading MYMD. The cryptic species of whitefly included Asia II 1 (dominant in Northern India), Asia II 8 (dominant in most of Southern, India) and Asia 1 (present in Hyderabad, Telangana, and Coimbatore, Tamil Nadu locations of Southern India). Gene pyramiding the incorporation of multiple resistant genes in a cultivar is seen as an alternative to breeding for diseases/insect resistance with several strains/biotypes.

Though there have been several continued attempts to evolve crop varieties/genotypes for a specific biotic and abiotic stress, on a larger scale, the success achieved was less owing to the combined impact of several stresses and unexpected sudden episodes of pests and diseases all along growth stages of the plants; hence, only a few countable successes have been reported in legumes, more so in cereals. Stemming the critical stage of crop growth for breeding itself need a thorough assessment, be seed germination, early vigour or field establishment, vegetative phase, flowering and early podding to podding stage, reproductive to final maturity stages etc. In this array of developmental stages, pinning down a specific stage and the very influencing trait for breeding seems very challenging though several strategies have hovered around flowering and reproductive phase (being termed `sensitive’) with an objective to develop breeding lines that withstand stress load and produce relatively better pod and seed yield.

## Future Outlook

Though a number of disease resistant lines have been developed for yellow mosaic, powdery mildew, and CLS, very few resistant sources are available for anthracnose, dry root rot and bacterial diseases. Further, molecular markers developed for powdery mildew and CLS need to be used in the breeding program to develop further disease resistant lines. Development of markers for dry root rot and anthracnose is needed to fast track development of disease resistant lines. Insect resistant sources of few insects such as bruchids and whiteflies are available, which are being used in breeding programs to develop insect resistant mungbean. However, there is every possibility of the introgression of undesired traits from these resistant sources to the cultivars. In order to have stable disease and insect resistant mungbean for a specific disease or pest, a synergy between the conventional breeding techniques and molecular technologies is very important (Kim et al., 2015; Schafleitner et al., 2016). Identification of molecular markers will help in the evaluation of the diseases and pest resistance and reduce our dependency on the phenotypic data, which might be laborious in big trials (Kitamura et al., 1988; Chen et al., 2007). Further, using molecular markers can help to transfer insect resistance from the related legumes such as black gram into mungbean. However, it is very important to identify and combine multiple resistant genes into the same cultivar. Thus gene pyramiding should be the target for breeders to develop mungbean with resistance to diseases and insect-pests and avoid strain/biotype development. The mechanism of diseases and insect resistance needs to be studied to identify herbivore- and pathogen-specific signal molecules and their mode of action. Furthermore, the RNAi technology can be used to improve biotic stress resistance in mungbean. However, in order to establish RNAi technology as a potential pest management strategy in plant breeding, large-scale field studies are essential. Further, the potential risks of this technology needs attention.

Breeding mungbean lines for stressful environments is very important. While in particular, stress dominates a population of environments, many of the agroecologies are featured by multiple stresses. This often makes a particular agro-ecology unique for which systemized solutions are essential. For making the best combination of abiotic stress and the traits to incorporate, it is essential to have insight on the fundamental mechanism for stress tolerance from intrinsic physiological and biochemical perspectives. We aim to develop root systems that help plants to withstand moisture deficits by drawing water from the deeper soils. Screening for various abiotic stresses needs to be more precise and stringent to identify robust donor/s for these traits. The identified donors need to put in use by the breeders at a faster pace. Plant type/s having a deep root system, early maturity span, erect stature with sympodial pod-bearing, multiple pods per cluster and longer pods with many nodes and shorter internodes will help in withstanding heat and drought-related stresses. Of late, converging various modern technologies like, infra-red thermography, automated robotics, camera images, and computational algorithms, which all make components of high throughput phenotyping facilities (phenomics and phenospex) can facilitate high throughput phenotyping for stress tolerance (Pratap et al., 2019b). However, non-destructive methods being utilized for targeted regions or environments needs optimization for establishing a relation between the known difficult to measure traits and the surrogate parameters derived from images, which represent plant responses to abiotic stresses. These phenomics methods can help precisely quantifying plant shoot architectural responses to stresses caused by soil moisture deficit, salinity, high temperature etc. More than a dozen image parameters have been explained to illustrate the responses of plants to stress that can guide in identifying the relevant traits and the protocol for screening large number of breeding lines or mapping population that are aiming at identification of stress tolerant genes. As evident from published literature, some of the traits such as high photosynthesis or quantum yields have been associated with tolerance to drought, salinity or high temperature. Generally, it is attributed to the capacity of plants to maintain water balance in the tissue reflected by relative water content and stress avoidance mechanism. However, it is essential to look into the traits such as capacity to retain physiological function, for example, even at 50% of optimum relative water content. Such traits are not feasible for application in plant breeding program with conventional approach. However, plant phenomics platform allow no destructive measurement of physiological function such as chlorphyll fluorescence based PS-II system. They are also equipped with NIR-based tools to assess non-destructively tissue water status in plants subjected to stress. These tools can allow measurement of tolerance of PS-II system health at given levels of tissue water content and hence true tolerance to stresses such as soil moisture deficit, salinity and high temperatures. Further, mechanisms to escape from abiotic stresses like drought and high temperatures are extensively been explored in many crops to get optimum yield in stress prone agroecologies. However, there is scope for exploring diurnal escape from stress in a way that plant can exhibit water saving mechanisms during peak stress hours in the diurnal cycle and keep their stomata open for sufficiently capture ambient CO_2_. It is possible to quantify such traits by strategically employing phenomics tools such as infrared imaging system. High temperatures during nights, is likely to enhance respiratory loss of assimilates, however, there are no mechanisms to measure these traits. It is essential to device tools/protocols for these measurements either in high or semi-throughput modes. Since mungbean is grown largely in marginal environments or in a short time between harvest and sowing of preceding and subsequent crops, it is essential to assess recovery from stress and performance in terms of seed yield. Continuous monitoring image based system can allow precise quantification of these traits by separating developmental changes from actual impact of stress. Recently evolved CT scan based tools and protocols will allow understand root-soil-water interaction and can quantify roots system architecture more precisely. This will open up new avenues for designing phenomics and genomics approaches for supporting improvement of stress tolerance in crops.

Molecular approaches are becoming handy in revealing resistance/tolerance mechanisms, which will help in modifying mungbean plants to suit the biotic and abiotic stresses. Genome Wide Association Studies [Noble et al., 2018; Breria et al., 2019)] would help in better understanding of the genetic basis of the phenotypes. Association mapping for biotic and abiotic resistant/tolerant traits is highly important to identify the desired haplotypes in performing association mapping on a panel of adapted elite breeding lines. This will provide the ample justification to utilize these lines directly in breeding programs. The selection of favorable haplotypes through MAS will be reduce the phenotyping material in the advanced breeding generations and increase the breeding efficiency. The development of NGS technologies, the discovery of SNP/alleles has become easy. This mungbean diversity panel constitutes a valuable resource for genetic dissection of important agronomic traits to accelerate mungbean breeding. Genetic variability with mungbean and between closely related species can be studied from the sequence-based information, which forms a pre-requisite criterion for breeding for resistant/tolerance to biotic and abiotic stress. This is also important for the species conservation and provides breeders with new and/or beneficial alleles for developing advanced breeding materials. Further, advanced phenotyping technologies such as NGS help to increase the discovery of trait-allele and genotype-phenotype interactions. There must be systematic efforts towards exploring physiological and biochemical regulations of biotic and abiotic stresses and studying the whole profile of genes, proteins and metabolites imparting resistance/tolerance so that the same can be manipulated to develop improved cultivars of mungbean.

## Author Contributions

RN—conceived the idea and contributed to the review in general. AKP and AW—contributed mainly to the biotic stress section. HB and JR—contributed mainly to the abiotic stress section. TS, AA, AP, SM, RK, EKM, CD, and RS contributed to the review in general.

## Funding

The financial assistances for this review was provided by Australian Center for International Agricultural Research (ACIAR) through the projects on International Mungbean Improvement Network (CIM-2014-079) and UKaid project on “Unleashing the economic power of vegetables in Africa through quality seed of improved varieties,” the strategic long-term donors to the World Vegetable Center: Republic of China (Taiwan), UK aid from the UK government, United States Agency for International Development (USAID), Germany, Thailand, Philippines, Korea, and Japan. Authors also thank ICAR-NICRA for supporting research on stress tolerance in mungbean.

## Conflict of Interest

The authors declare that the research was conducted in the absence of any commercial or financial relationships that could be construed as a potential conflict of interest.

## References

[B1] Abate T. Girma A. Ayalew G. (1995). Progress in host plant resistance research against bean stem maggots. Afr. Crop Sci. Conf. Proc. 2, 167–173.

[B2] Abdullah-Al-Rahad M. Rahman M. S. Akter T. Akter J. Rahman M. A. Aziz S. M. S. (2018). Varietal screening of mungbean against whitefly and aphid. J. Biosci. Agric. Res. 18, 1478–1487. 10.18801/jbar.180118.183

[B3] Abhary M. Rezk A. (2015). “RNAi technology: a potential tool in plant breeding,” in Advances in Plant Breeding Strategies: Breeding, Biotechnology and Molecular Tools. Eds. Al-Khayri J. M. Jain S. M. Johnson D. V. (Springer publishers), 397–427. 10.1007/978-3-319-22521-0_14

[B4] ACIAR (Australian Center for International Agricultural Research) (2019). Mung Central ed. 05, July 2019. pp 7. https://www.aciar.gov.au/publication/Mung-Central-Newsletter-edition-five (Retreived on 20 August 2019).

[B5] ACIAR (Australian Center for International Agricultural Research) (2018). Mung Central ed. 04, July 2018. pp 5. https://www.aciar.gov.au/publication/Mung-Central-edition-four (Retreived on 20 August 2019).

[B6] Acosta-Gallegos J. A. Kelly J. D. Gepts P. (2008). Prebreeding in common bean and use of genetic diversity from wild germplasm. Crop Sci. 48, 3–16. 10.2135/cropsci2007.04.0008IPBS

[B7] Ahmad P. Prasad M. N. V. (2012). Abiotic Stress Responses in Plants: Metabolism, Productivity and Sustainability. New York, NY: Springer. 10.1007/978-1-4614-0634-1

[B8] Ahmed S. (2009). Effect of soil salinity on the yield and yield components of mungbean. Pak. J. Bot. 41 (1), 263–268.

[B9] Ahmed F. Rafii M. Y. Ismail M. R. Juraimi A. S. Rahim H. A. Asfaliza R. (2013). Waterlogging tolerance of crops: breeding, mechanism of tolerance, molecular approaches, and future prospects. BioMed. Res. Int. 10 (963525) 10.1155/2013/963525 PMC3591200 23484164

[B10] Akhtar J. Lal H. C. Kumar Y. Singh P. K. Ghosh J. Khan Z. (2014). Multiple disease resistant in greengram and blackgram germplasm and management through chemicals under rain-fed conditions. Leg. Res. 37, 101–109. 10.5958/j.0976-0571.37.1.016

[B11] Akhtar K. P. Kitsanachandee R. Srinives P. Abbas G. Asghar M. J. Shah T. M. (2009). Field evaluation of mungbean recombinant inbred lines against mungbean yellow mosaic disease using new disease scale in Thailand. Plant Pathol. J. 25, 422–428. 10.5423/PPJ.2009.25.4.422

[B12] Akhtar K. P. Sarwar G. Abbas. G. Asghar M. J. Sarwar N. Shah T. M. (2011). Screening of mungbean germplasm against mungbean yellow mosaic India virus and its vector *Bemisia tabaci*. Crop Prot. 30, 1202–1209. 10.1016/j.cropro.2011.05.012

[B13] Alam A. K. M. M. Somta P. Srinives P. (2014). Identification and confirmation of quantitative trait loci controlling resistance to mungbean yellow mosaic disease in mungbean [*Vigna radiata* (L.) Wilczek]. Mol. Breed. 34, 1497–1506. 10.1007/s11032-014-0133-0

[B14] Ali M. Malik I. A. Sabir H. M. Ahmad B. (1997). “The Mungbean Green Revolution in Pakistan,” in Technical Bulletin No. 24. AVRDC (Shanhua, Taiwan: ROC), 66.

[B15] Almeida N. F. Leitão S. T. Krezdorn N. Rotter B. Winter P. Rubiales D. (2014). Allelic diversity in the transcriptomes of contrasting rust-infected genotypes of *Lathyrus sativus*, a lasting resource for smart breeding. BMC Plant Biol. 14, 376. 10.1186/s12870-014-0376-2 25522779 PMC4331309

[B16] Ambede J. G. Netondo G. W. Mwai G. N. Musyimi D. M. (2012). NaCl salinity affects germination, growth, physiology, and biochemistry of bambara groundnut. Braz. J. Plant Physiol. 24, 151–160. 10.1590/S1677-04202012000300002

[B17] Ammavasai S. Phogat D. S. Solanki I. S. (2004). Inheritance of resistance to mungbean yellow mosaic virus (MYMV) in green gram (*Vigna radiata* L Wilczek). Ind. J. Genet. 64, 145–146.

[B18] Anderson T. R. (1985). Root rot and wilt of mungbean in Ontario. Can. Plant Dis. Surv. 65, 3–6.

[B19] Anjum N. A. Umar S. Aref I. M. Iqbal M. (2015). Managing the pools of cellular redox buffers and the control of oxidative stress during the ontogeny of drought-exposed mungbean (*Vigna radiata* L.)—role of sulfur nutrition. Front. Environ. Sci. 2, 66. 10.3389/fenvs.2014.00066

[B20] Arnell N. Liu C. (2001). “Climatic Change 2001: hydrology and water resources,” in Report from the Intergovernmental Panel on Climate Change. Available at http://www.ipcc.ch/. (Verified on January 7th 2019).

[B21] Arun K. V. Venkateswarlu B. (2011). Abiotic stress in Plants – Mechanisms and Adaptations. INTECHWEB.org, 1–440.

[B22] Asensio-S. Manzanera M. C. Asencio C. Singh S. P. (2006). Gamete selection for resistance to common and halo bacterial blights in dry bean intergene pool population. Crop Sci. 46, 131–135. 10.2135/cropsci2005.0198

[B23] Asensio-S. Manzanera M. C. Asencio C. Singh S. P. (2005). Introgressing resistance to bacterial and viral diseases from the Middle American to Andean common bean. Euphytica 143, 223–228. 10.1007/s10681-005-3860-9

[B24] AVRDC (1979). AVRDC Progress Report for (1978). Shanhua, Tainan: Asian Vegetable Research and Development Center, 173.

[B25] AVRDC (1987). 1984 Progress Report. Shanhua, Tainan: Asian Vegetable Research and Development Center, 480.

[B26] AVRDC (1988). 1986 Progress Report. Shanhua, Tainan: Asian Vegetable Research and Development Center, 540.

[B27] AVRDC (1991). 1990 Progress Report. Shanhua, Tainan: Asian Vegetable Research and Development Center, 312.

[B28] Bailey-Serres J. Voesenek L. A. (2008). Flooding stress: acclimations and genetic diversity. Ann. Rev. Plant Biol. 59, 313–339. 10.1146/annurev.arplant.59.032607.092752 18444902

[B29] Basak J. Kundagrami S. Ghose T. K. Pal A. (2004). Development of Yellow Mosaic Virus (YMV) resistance linked DNA marker in *Vigna mungo* from populations segregating for YMV-reaction. Mol. Breed. 14, 375–383. 10.1007/s11032-004-0238-y

[B30] Bashir M. Ahmad Z. Ghafoor A. (2005). Sources of genetic resistance in mungbean and blackgram against urdbean leaf crinkle virus (ULCV). Pak. J. Bot. 37, 47–51.

[B31] Bashir M. Malik B. A. (1988). Diseases of major pulse crops in Pakistan—a review. Trop. Pest. Manag. 34, 309–314. 10.1080/09670878809371262

[B32] Beck E. H. Fetitig S. Knake C. Hartig K. Bhattarai T. (2007). Specific and unspecific responses of plants to cold and drought stress. J. Bio. Sci. 32, 501–510. 10.1007/s12038-007-0049-5 17536169

[B33] Begg J. E. (1980). “Morphological adaptation of leaves to water stress,” in Adaptation of plants to water and high temperature stress. Eds. Turner N. C. Kramer P. J. (New York: John Wiley and Sons).

[B34] Bhandari K. Kamal D. S. Bindumadhava H. Kadambot H. M. S. Gaur P. Shiv Kumar A. (2017). Temperature sensitivity of Food Legumes: A Physiological insight. Acta Physiol. Plant 3968, 1–22. 10.1007/s11738-017-2361-5

[B35] Bhaskar A. V. (2017). Genotypes against major diseases in green gram and black gram under natural field conditions, A. Vijaya Bhaskar. Int. J. Curr. Microbiol. App. Sci. 6, 832–843. 10.20546/ijcmas.2017.606.098

[B36] Bhat F. A. Mohiddin F. A. Bhat H. A. (2014). Reaction of green gram (*Vigna radiata*) to *Cercospora canascens* (ELL.) and Mart. Ind. J. Agric. Res. 48, 140–144. 10.5958/j.0976-058X.48.2.023

[B37] Bhople S. K. Dhandge S. R. Aravindarajan G. Patange N. R. (2017). Varietal screening of mungbean genotypes for their resistance against pest complex of mungbean. AGRES 6, 123–128.

[B38] Bindumadhava H. Sharma L. Nair R. M. Nayyar H. Riley J. J. Easdown W. (2018). High-temperature-tolerant mungbean (*Vigna radiata* L.) lines produce better yields when exposed to higher CO2 levels. J. Crop Improv. 32, 418–430. 10.1080/15427528.2018.1439132

[B39] Binyamin R. Khan M. A. Khan N. A. Khan A. I. (2015). Application of SCAR markers linked with mungbean yellow mosaic virus disease-resistance gene in Pakistan mungbean Germplasm. Gen. Mol. Res. 14, 2825–2830. 10.4238/2015.March.31.13 25867432

[B40] Biswas J. C. Kalra N. Maniruzzaman M. Choudhury A. K. Jahan M. A. H. S. Hossain M. B. (2018). Development of mungbean model (MungGro) and its application for climate change impact analysis in Bangladesh. Ecol. Modell. 384, 1–9. 10.1016/j.ecolmodel.2018.05.024

[B41] Blair M. W. Muñoz C. Buendía H. F. Flower J. Bueno J. M. Cardona C. (2010). Genetic mapping of microsatellite markers around the arcelin bruchid resistance locus in common bean. Theor. Appl. Genet. 121, 393–402. 10.1007/s00122-010-1318-5 20358173 PMC2886137

[B42] Blair M. W. Muñoz C. Garca R. Cardona C. (2006). Molecular mapping of genes for resistance to the bean pod weevil (*Apion godmani* Wagner) in common bean. Theor. Appl. Genet. 112, 13–923. 10.1007/s00122-005-0195-9 16397789

[B43] Boyer J. S. Byrn P. Cassman K. G. Cooper M. Delmer D. Greene T. (2013). The U.S. drought of 2012 in perspective: A call to action. Glob. Food Secur. 2, 139–143. 10.1016/j.gfs.2013.08.002

[B44] Boykin L. M. De Barro P. (2014). A practical guide to identifying members of the *Bemisia tabaci* species complex: and other morphologically identical species. Front. Ecol. Evol. 2, 45. 10.3389/fevo.2014.00045

[B45] Breria C. M. Hsieh C. H. Yen J. Y. Nair R. Lin C.-Y. Huang S.-M. (2019). Population SStructure of the World Vegetable Center mungbean mini core collection and Genome-Wide Association mapping of loci associated with variation of seed coat luster Trop. Plant Biol. 10.1007/s12042-019-09236-0

[B46] Brumfield R. T. Beerli P. Nickerson D. A. Edwards S. V. (2003). The utility of single nucleotide polymorphisms in inferences of population history. Trends Ecol. Evol. 18, 249–256. 10.1016/S0169-5347(03)00018-1

[B47] Burton A. Widstorm N. W. (2001). Mass selection for agronomic performance and resistance to ear feeding insects in three corn populations. Maydica 46, 207–212.

[B48] Chaitieng B. Kaga A. Han O. K. Wang X. Wongkaew S. Laosuwan P. (2002). Mapping a new source of resistance to powdery mildew in mungbean. Plant Breed. 121, 521–525. 10.1046/j.1439-0523.2002.00751.x

[B49] Chand R. Singh V. Pal C. Kumar P. Kumar M. (2012). First report of a new pathogenic variant of *Cercospora canescens* on mungbean (*Vigna radiata*) from India. New Dis. Rep. 26, 6. 10.5197/j.2044-0588.2012.026.006

[B50] Chankaew S. Somta P. Isemura T. Tomooka N. Kaga A. Vaughan D. A. (2013). Quantitative trait locus mapping reveals conservation of major and minor loci for powdery mildew resistance in four sources of resistance in mungbean [*Vigna radiata* (L.) Wilczek]. Mol. Breed. 32, 121–130. 10.1007/s11032-013-9856-6

[B51] Chankaew S. Somta P. Sorajjapinun W. Srinives P. (2011). Quantitative trait loci mapping of Cercospora leaf spot resistance in mungbean, *Vigna radiata* (L.) Wilczek. Mol. Breed. 28, 255–264. 10.1007/s11032-010-9478-1

[B52] Chauhan R. Singh A. K. Sharma K. R. Ali A. (2018). Screening of mungbean (*Vigna radiata* L.) germplasm against major sucking pest. J. Pharm. Phytochem. 7, 1784–1787.

[B53] Chauhan Y. S. Douglas C. Rachaputi R. C. N. Agius P. Martin W. King K. (2010). “Physiology of mungbean and development of the mungbean crop model,” in Proceedings of the 1st Australian Summer Grains Conference Australia, Gold Coast, QL 21–24.

[B54] Chen H. M. Ku H. M. Schafleitner R. Bains T. S. Kuo C. G. Liu C. A. (2012). The major quantitative trait locus for Mungbean yellow mosaic Indian virus resistance is tightly linked in repulsion phase to the major bruchid resistance locus in a cross between mungbean [*Vigna radiata* (L.) Wilczek] and its wild relative *Vigna radiata* ssp. . Euphytica 192, 205–216. 10.1007/s10681-012-0831-9

[B55] Chen H. M. Ku H. S. Schafleitner R. Bains T. S. Kuo G. C. Liu C. A. (2013). The major quantitative trait locus for *mungbean yellow mosaic Indian virus* resistance is tightly linked in repulsion phase to the major bruchid resistance locus in a cross between mungbean [*Vigna radiata* (L.) Wilczek] and its wild relative *Vigna radiata* ssp. sublobata. Euphytica 192, 205–216. 10.1007/s10681-012-0831-9

[B56] Chen H. M. Liu C. A. Kuo C. G. Chien C. M. Sun H. C. Huang C. C. (2007). Development of a molecular marker for a bruchid (*Callosobruchus chinensis* L.) resistance gene in mungbean. Euphytica 157, 113–122. 10.1007/s10681-007-9400-z

[B57] Chhabra K. S. Kooner B. S. (1998). “Insect pest management in mungbean and blackgram- status and strategies,” in Pulses. IPM system in agriculture, vol. 4 Eds. Upadhyay R. Mukerji K. G. Rajak R. L. (New Delhi: Aditya Books Publishing Pvt. Ltd), 233–310.

[B58] Chhabra K. S. Kooner B. S. Sharma A. K. Saxena A. K. Shanmugasundaran S. (1988). Mungbean Proceedings of Second International Symposium. Bangkok, Thailand, 16–22.

[B59] Chiang H. S. Talekar N. S. (1980). Identification of sources of resistance to beanfly and two other agromyzid flies in soybean and mungbean. J. Econ. Entomol. 73, 197–199. 10.1093/jee/73.2.197

[B60] Chiel E. Gottlieb Y. Zchori-Fein E. Mozes-Daube N. Katzir N Inbar M. (2007). Biotype-dependent secondary symbiont communities in sympatric populations of *Bemisia tabaci*. Bull. Entomol. Res. 97, 407–413. 10.1017/S0007485307005159 17645822

[B61] Chotechung S. Somta P. Chankaew S. Srinives P. Somta P. (2011). Identification of DNA markers associated with bruchid resistance in mungbean. Khon Kaen Agri. J. 39, 221–226. 10.1186/s12870-016-0847-8

[B62] Chotechung S. Somta P. Chen J. Yimram T. Chen X. Srinives P. (2016). A gene encoding a polygalacturonase–inhibiting protein (PGIP) is a candidate gene for bruchid (Coleoptera: bruchidae) resistance in mungbean (*Vigna radiata*). Theor. Appl. Genet. 129, 1673–1683. 10.1007/s00122-016-2731-1 27220975

[B63] Choudhary S. Choudhary A. K. Sharma O. P. (2011). Screening of mungbean (*Vigna radiata*) genotypes to identify source of resistant to dry root rot. J. Food Leg. 24, 117–119.

[B64] Cramer G. R. Urano K. Delrot S. Pezzotti M. Shinozaki K. (2011). Effects of abiotic stress on plants: a systems biology perspective. BMC Plant Biol. 11, 163. 10.1186/1471-2229-11-163 22094046 PMC3252258

[B65] Dabrowski Z. T. Bungu D. O. M. Ochieng R. S. (1983). Studies on the legume pod-borer, *Maruca testulalis* (Geyer) Methods used in cowpea screening for resistance. Insect Sci. Appl. 4, 141–145. 10.1017/S1742758400004148

[B66] Dennis J. R. Howard J. Vogel V. (1999). Molecular shuttles: directed motion of microtubules along nanoscale kinesin tracks. Nanotechnology 10, 232–236. 10.1088/0957-4484/10/3/302

[B67] Devasthali S. Joshi M. (1994). Infestation and varietal preference of insect-pests in green gram. Ind. Agric. 38, 263–272.

[B68] Dhillon N. P. S. Wehner T. C.(1991). Host-plant resistance to insects in cucurbits - germplasm resources, genetics and breeding. Trop. Pest. Manage. 37, 421–428. 10.1080/09670879109371628

[B69] Dhole V. J. Reddy K. S. (2012). Genetic analysis of resistance to mungbean yellow mosaic virus in mungbean (*Vigna radiata*). Plant Breed. 131, 414–417. 10.1111/j.1439-0523.2012.01964.x

[B70] Dinesh H. B. Lohithaswa H. C. Viswanatha K. P. Singh P. Rao A. M. (2016). Identification and marker-assisted introgression of QTL conferring resistance to bacterial leaf blight in cowpea (*Vigna unguiculata* (L.) Walp.). Plant Breed. 135, 506–512. 10.1111/pbr.12386

[B71] Distabanjong K. P. Srinives P. (1985). Inheritance of beanfly resistance in mungbean (*Vigna radiata* (L.) Wilczek. Kasetsart J. Nat. Sci. 19, 75–84.

[B72] Dubrovina A. S. Kiselev K. V. (2019). Exogenous RNAs for gene regulation and plant resistance. Int. J. Mol. Sci. 20, 2282. 10.3390/ijms20092282 PMC6539981 31072065

[B73] Dutta P. Bandopadhyay P. Bera A. K. (2016). Identification of Leaf based physiological markers for drought susceptibility during early seedling development of mungbean. Am. J Plant Sci. 7, 1921–1936. 10.4236/ajps.2016.714176

[B74] Dutta P. Bera A. K. (2008). Screening of mungbean genotypes for drought tolerance. Leg. Res. 31, 145–148.

[B75] Edwards O. Singh K. B. (2006). Resistance to insect-pests: what do legumes have to offer? Euphytica 147, 273–285. 10.1007/s10681-006-3608-1

[B76] Fathy N. E. Ismail S. M. Basahi J. M. (2018). Optimizing mungbean productivity and irrigation water use efficiency through the use of low water- consumption during plant growth stages. Legume Res. 41, 108–113.

[B77] Fernandez G. C. J. Kuo C. G. (1993). “Effective selection criteria for assessing plant stress tolerance,” in Adaptation of Food Crops to Temperature and Water Stress. Ed. Kuo C. G., Proceedings of the International Symposium, August 13-18 (1992) Tainan, Taiwan 257–270.

[B78] Fernandez G. C. J. Shanmugasundaram S. (1988). “The AVRDC Mungbean Improvement Program: The Past, Present and Future,” in Mungbean. Eds. Shanmugasundaram S. McLean B. T., Proceedings of the Second International Symposium held at Bangkok, Thailand. 58–70.

[B79] Fiorani F. Schurr U. (2013). Future scenarios for plant phenotyping. Annu. Rev. Plant Biol. 64, 267–291. 10.1146/annurev-arplant-050312-120137 23451789

[B80] Flowers T. (2006). Preface: ‘Special Issue: Plants and salinity. J. Exp. Bot. 57, 4. 10.1093/jxb/erj119

[B81] Frei A. Blair M. W. Cardona C. Beebe S. E. Gu H. Dorn S. (2005). QTL mapping of resistance to *Thrips palmi* Karny in common bean. Crop Sci. 45, 379–387. 10.2135/cropsci2005.0379

[B82] Fujii K. Miyazaki S. (1987). Infestation resistance of wild legumes (*Vigna sublobata*) to azuki bean weevil, *Callosobruchus chinensis* (L.) (Coleoptera: Bruchidae) and its relationship with cytogenetic classification. Appl. Entomol. Zool. 22, 319–322. 10.1303/aez.22.229

[B83] Fujii K. Ishimoto M. Kitamura K. (1989). Patterns of resistance to bean weevils (Bruchidae) in *Vigna radiata-mungo sublobata* complex inform the breeding of new resistant variety. Appl. Ent. Zool. 24, 126–132. 10.1303/aez.24.126

[B84] Gangwar B. Ahmed R. (1991). Performance of mungbean varieties under andaman and nicobar island condition. Ind. J. Pulse Res. 4, 115–116.

[B85] Goyary D. (2009). Transgenic crops, and their scope for abiotic stress environment of high altitude: biochemi- cal and physiological perspectives. DRDO Sci. Spectr., March 2009 195–201.

[B86] Hammer G. L. Oosterom E. V. McLean G. Chapman S. C. Broad I. Peter H. (2010). Adapting APSIM to model the physiology and genetics of complex adaptive traits in field crops. J. Expl. Bot. 61, 2185–2202. 10.1093/jxb/erq095 20400531

[B87] HanumanthaRao B. Nair R. M. Nayyar H. (2016). Salinity and high temperature tolerance in mungbean [*Vigna radiata* (L.) Wilczwk] from a physiological perspective. Front. Plant Sci. 7, 1–20. 10.3389/fpls.2016.00957 27446183 PMC4925713

[B88] Haq Q. M. I. Ali A. Malathi V. G. (2010). Engineering resistance against Mungbean yellow mosaic India virus using antisense RNA. Ind. J. Virol. 21, 82–85. 10.1007/s13337-010-0003-2 PMC3550775 23637483

[B89] Hartman G. L. Wang T. C. Kim D. (1993). Field evaluation of mungbeans for resistance to Cercospora leaf spot and powdery mildew. Int. J. Pest. Manag. 39, 418–421. 10.1080/09670879309371833

[B90] He X. He T. Xiong Y. Jiao C. (1988). “Research and use of mungbean germplasm resources in Hubei, China,” in Mungbean. Eds. Fernandez J. Shanmugsundaram S. (Shanhua, Tainan: Asian Vegetable Research and Development Centre), 35–41.

[B91] Holeyachi P. Savithramma D. L. (2013). Identification of RAPD markers linked to MYMV resistance in mungbean (*Vigna radiata* (L). Wilczek). Biosacn. J. 8, 1409–1411.

[B92] Hong M. G. Kim K. H. Ku J. H. Jeong J. K. Seo M. J. Park C. H. (2015). Inheritance and quantitative trait loci analysis of resistance genes to bruchid and bean bug in mungbean (*Vigna radiata* L. Wilczek). Plant Breed. Biotechnol. 3, 39–46. 10.9787/PBB.2015.3.1.039

[B93] Hossain M. Hamid A. Khaliq M. (2010). Evaluation of mungbean (*Vigna radiata* L.) genotypes on the basis of photosynthesis and dry matter accumulation. J. Agric. Rural Dev. 7, 1–8. 10.3329/jard.v7i1.4415

[B94] Humphry M. E. Konduri V. Lambrides C. J. Magner T. McIntyre C. L. Aitken E. A. B. (2002). Development of a mungbean (*Vigna radiata*) RFLP linkage map and its comparison with lablab (*Lablab purpureus*) reveals a high level of collinearity between the two genomes. Theor. Appl. Genet. 105, 160–166. 10.1007/s00122-002-0909-1 12582573

[B95] Humphry S. M. E. Magner T. McIntyre C. L. Aitken E. A. Liu C. L. (2003). Identification of major locus conferring resistance to powdery mildew (*Erysiphe polygoni* D.C.) in mungbean (*Vigna radiata* L. Wiczek) by QTL analysis. Genome 46, 738–744. 10.1139/g03-057 14608390

[B96] Huynh B. Jeffrey D. E. Arsenio N. Wanamaker S. Lucas M. R. Close T. J. (2015). Genetic mapping and legume synteny of aphid resistance in African cowpea (*Vigna unguiculata* L. Walp.) grown in California. Mol. Breed. 35, 36. 10.1007/s11032-015-0254-0 25620880 PMC4300395

[B97] Iqbal S. M. Ghafoor A. Bashir M. Malik B. A. (1995). Estimation of losses in yield components of mugbean due to *Cercospora* leaf spot. Pak. J. Phytopathol. 7, 80–81.

[B98] Iqbal S. M. Zubair M. Haqqani A. M. (2004). Resistant in Mungbean to *Cercospora* leaf spot disease. Int. J. Agric. Biol. 06, 792–793.

[B99] Iqbal S. M. Zubair M. Anwar M. Haqqani A. M. (2003). Resistance in mungbean to bacterial leaf spot disease. Mycopath 1, 81–83.

[B100] Iqbal S. M. Zubair M. Hussain S. Malik B. A. (1991). Reaction of mungbean genotypes to bacterial leaf spot disease. Pak. J. Phytopathol. 3, 19–21.

[B101] Iqbal U. Iqbal S. M. Afzal R. Jamal A. Farooq M. A. Zahid A. (2011). Screening of mungbean germplasm against Mungbean yellow mosaic virus (MYMV) under field conditions. Pak. J. Phytopathol. 23, 48–51.

[B102] Itoh T. Garcia R. N. Adachi M. Maruyama Y. Tecson-Mendoza E. M. Mikami B. (2006). Structure of 8Sα globulin, the major seed storage protein of mung bean. Acta Crystallogr. D. Biol. Crystallogr. 62, 824–832. 10.1107/S090744490601804X 16790939

[B103] Jain R. Lavanya G. R. Reddy P. A. Babu G. S. (2013). Genetic inheritance of yellow mosaic virus resistance in mungbean [*Vigna radiata* (L.) Wilczek]. Trends Biosci. 6, 305–306.

[B104] James R. A. Blake C. Byrt C. S. Munns R. (2011). Major genes for Na+ exclusion, Nax1 and Nax2 wheatHKT1;4 and HKT1;5), decrease Na+ accumulation in bread wheat leaves under saline and waterlogged conditions. J. Exp Bot. 62, 2939–2947. 10.1093/jxb/err003 21357768

[B105] Kaewwongwal A. Chen J. Somta P. Kongjaimun A. Yimram T. Chen X. (2017). Novel Alleles of Two Tightly Linked Genes Encoding Polygalacturonase-Inhibiting Proteins (VrPGIP1 and VrPGIP2) Associated with the Br Locus that Confer Bruchid (Callosobruchus spp.) Resistance to Mungbean (*Vigna radiata*) Accession V2709. Front. Plant Sci. 8, 1692. 10.3389/fpls.2017.01692 29033965 PMC5625325

[B106] Kalaria R. K. Chauhan D. Mahatma M. K. Mahatma L. (2014). Identification of RAPD and ISSR makers for resistance against Mungbean Yellow Mosaic Virus in mungbean (*Vigna radiata* L.) under south Gujarat agro climatic condition of India. Bioscan 9, 1177–1182.

[B107] Karthikeyan A. Shobhana V. G. Sudha M. Raveendran M. Senthil N. Pandiyan M. (2014). Mungbean yellow mosaic virus (MYMV): a threat to green gram (Vigna radiata) production in Asia. Int. J. Pest. Manag. 60, 314–324. 10.1080/09670874.2014.982230

[B108] Kasettranan W. Somta P. Srinives P. (2009). Genetics of the resistance to powdery mildew disease in mungbean (*Vigna radiata* (L.) Wilczek). J Crop Sci. Biotechnol. 12, 37–42. 10.1007/s12892-008-0074-4

[B109] Kasettranan W. Somta P. Srinives P. (2010). Mapping of quantitative trait loci controlling powdery mildew resistance in mungbean (*Vigna radiata* (L.) Wilczek). J. Crop Sci. Biotechnol. 13, 155–161. 10.1007/s12892-010-0052-z

[B110] Kaur L. Singh P. Sirari A. (2011). Biplot analysis for locating multiple disease resistant diversity in mungbean germplasm. Dis. Res. 26, 55–60.

[B111] Kaur R. Bains T. S. Bindumadhava H. Nayyar H. (2015). Responses of mungbean (*Vigna radiata* L.) genotypes to heat stress: Effects on reproductive biology, leaf function and yield traits. Sci. Hort. 197, 527–541. 10.1016/j.scienta.2015.10.015

[B112] Kaur R. Kaur J. Bains T. S. (2017). Screening of mungbean genotypes for drought tolerance using different water potential levels. J. Adv. Agric. Tech. 4, 2–18. 10.18178/joaat.4.2.159-164

[B113] Kaushik C. D. Chand J. N. (1987). Seedborne nature of *Rhizoctonia bataticola* causing leaf blight of mungbean. J. Mycol. Plant Pathol. 17, 154–157.

[B114] Kelly J. Kolkman J. M. Schneider K. (1998). Breeding for yield in dry bean (*Phaseolus vulgaris* L.). Euphytica 102, 343–356. 10.1023/A:1018392901978

[B115] Kelly J. D. Adams M. W. (1987). Phenotypic recurrent selection in ideotype breeding of pinto beans. Euphytica 36, 69–80. 10.1007/BF00730649

[B116] Keneni G. Bekele E. Getu E. Imtiaz M. Damte T. Mulatu B. (2011). Breeding food legumes for resistance to storage insect-pests: potential and limitations. Sustainability 3, 1399–1415. 10.3390/su3091399

[B117] Khajudparn P. Wongkaew S. Thipyapong P. (2007). Mungbean powdery resistant identification of genes for resistant to powdery mildew in mungbean. Afr. Crop Sci. Conf. Proc. 8, 743–745.

[B118] Khan K. S. H. Shuaib M. (2007). Identification of sources of resistant in mungbean (*Vigna radiata* L.) against charcoal rot *Macrophomina phaseolina* (Tassi) Goid. Afr. Crop Sci. Conf. Proc. 8, 2101–2102.

[B119] Khattak M. K. Shafqat A. Chisti J. I. (2004). Varietal resistance of mungbean (*Vigna radiata* L.) against whitefly (*Bemisia tabaci* Genn.), jassid (*Amrasca devastans* Dist.), and thrips (*Thrips tabaci* Lind.). Pak. Entomol. 26, 9–12.

[B120] Khattak G. S. S. Haq M. A. Ashraf M. Elahi T. (2000). Genetics of Mungbean Yellow Mosaic Virus (MYMV) in mungbean (*Vigna radiata* L.) Wilczek. J. Genet.Breed. 54, 237–243.

[B121] Khattak G. S. S. Saeed I. Muhammad T. (2009). Flowers shedding under high temperature in mungbean (*Vigna radiata* (L.) Wilczek). Pak. J. Bot. 41, 35–39.

[B122] Kim S. K. Nair R. M. Lee J. Lee S. H. (2015). Genomic resources in mungbean for future breeding programs. Front. Plant Sci. 6, 626. 10.3389/fpls.2015.00626 26322067 PMC4530597

[B123] Kitamura K. Ishimoto M. Sawa M. (1988). Inheritance of resistance to infestation with azuki bean weevil in *Vigna sublobata* and successful incorporation to *V. radiata*. Jpn. J. Breed. 38, 459–464. 10.1270/jsbbs1951.38.459

[B124] Kitsanachandee R. Somta P. Chatchawankanphanich O. Akhtar P. Shah T. M. Nair R. M. (2013). Detection of quantitative trait loci for mungbean yellow mosaic India virus (MYMIV) resistance in mungbean (*Vigna radiata*(L.) Wilczek) in India and Pakistan. Breed. Sci. 63, 367–373. 10.1270/jsbbs.63.367 24399908 PMC3859347

[B125] Kooner B. S. Chhabra K. S. Arora B. S. (1997). Resistant sources in mungbean to manage whitefly, jassids and yellow mosaic virus. In: Proceedings of third agricultural science congress March 12- 15, PAU Ludhiana, India 2.

[B126] Kooner B. S. Cheema H. K. (2007). Screening of mungbean germplasm against whitefly, *Bemisia tabaci* and MYMV. Acta Hortic. 752, 307–310. 10.17660/ActaHortic.2007.752.52

[B127] Kulkarni S. A. Epidemiology and integrated management of anthracnose of green gram, (2009), M.Sc. (Agri.) Thesis submitted to UAS Dharwad, Karnataka. 1–170

[B128] Kumar R. Singh P. S. (2017). Screening of certain mungbean, *Vigna radiata* (L.) Wilczek genotypes against spotted pod borer and pod bugs. J. Exp. Zool. Ind 1, 595–597.

[B129] Kumar A. Sharma K. D. (2009). Physiological responses and dry matter partitioning of summer mungbean (*Vigna radiata* L.) genotypes subjected to drought conditions. J. Agron. Crop Sci. 95, 270–277. 10.1111/j.1439-037X.2009.00373.x

[B130] Kumar J. Doshi A. Epidemiology and management of bacterial leaf spot of green gram [Vigna radiata (L.) Wilczek] caused by Xanthomonas axonopodis pv. vigna radiata (Sabet et al.) Dye, (2016), PHD Thesis, MPUAT, Udaipur Pp-151.

[B131] Kumar S. Tanti B. Patil B. L. Mukherjee S. K. Sahoo L. (2017). RNAi-derived transgenic resistance to *Mungbean yellow mosaic India virus* in cowpea. PLoS One 12, e0186786. 10.1371/journal.pone.0186786 29077738 PMC5659608

[B132] Lal S. S. (1987). “Insect-pests of mungbean, urd, cowpea, and pea and their management,” in Plant protection in field crops. Eds. Rao V. M. Sithanantham S. (Hyderabad: Plant Protection Association of India), 185–202.

[B133] Lamichaney A. Katiyar P. Laxmi V. Pratap A. (2017). Variation in pre-harvest sprouting tolerance and fresh seed germination in mungbean (*Vigna radiata* L.) genotypes. Plant Genet. Resour.: Charact. Util. 16, 437–445. 10.1017/S1479262117000296

[B134] Lamseejan S. Smutkupt S. Wongpiyasatid A. Naritoom K. (1987). Use of Radiation in Mungbean Breeding, In: Mungbean Proceedings of the Second, International Symposium, Nov. 16-20, Bangkok p.174–177.

[B135] Latif M. Akram N. A. Ashraf M. (2016). Regulation of some biochemical attributes in drought-stressed cauliflower (*Brassica oleracea* L.) by seed pre-treatment with ascorbic acid. J. Hort. Sci. Biotechnol. 91, 129–137. 10.1080/14620316.2015.1117226

[B136] Lawn R. J. Williams R. W. Imrie B. C. (1988). “Potential of wild germplasm as a source of tolerance to environmental stresses in mungbean,” in Mungbean. Eds. Fernandez J. Shanmugsundaram S. (Shanhua, Tainan: Asian Vegetable Research and Development Centre), 136–145.

[B137] Lee Y. B. (1980). Inheritance study on resistance to Cercospora leaf spot in mungbean. Shanhua, Taiwan: Asian Vegetable Research and Development Center.

[B138] Liu L. Li Y. Li S. Hu N. He Y. Pong R. (2012). Comparison of next-generation sequencing systems. J. Biomed. Biotechnol., Article ID 251364. 11. 10.1155/2012/251364 22829749 PMC3398667

[B139] Liu M. S. Kuo T. C. Y. Ko C. Y. Wu D. C. Li K. Y. Lin W. J. (2016). Genomic and transcriptomic comparison of nucleotide variations for insights into bruchid resistance of mungbean (*Vigna radiata* [L.] R. Wilczek). BMC Plant Biol. 16, 46. 10.1186/s12870-016-0736-1 26887961 PMC4756517

[B140] Mahalingam A. Satya V. K. Manivannan N. Narayanan S. L. Sathya P. (2018). Inheritance of mungbean yellow mosaic virus disease resistance in greengram [*Vigna radiata* (L.) Wilczek]. Int. J. Curr. Microbiol. App. Sci. 7, 880–885. 10.20546/ijcmas.2018.701.107

[B141] Maheshwari S. K. Krishna H. (2013). Field efficacy of fungicides and bio-agents against *Alternaria* leaf spot of mungbean. Ann. Plant Prot. Sci. 21, 364–367.

[B142] Maiti S. Basak J. Kundagrami S. Kundu A. Pal A. (2011). Molecular marker-assisted genotyping of mungbean yellow mosaic India virus resistant germplasms of mungbean and urdbean. Mol. Biotechnol. 47, 95–104. 10.1007/s12033-010-9314-1 20652447

[B143] Malik S. P. S. Comparative resistance of summer mungbean genotype to the thrips, Megalurothrips distalis (Karny.), M.Sc. thesis, (1990)Punjab Agricultural University, Ludhiana.

[B144] Maliwal G. L. Paliwal K. V. (1982). Salt tolerance of some mungbean (*Vigna radiata*), urdbean (*Vigna mungo*) and guar (*Cyamopsis tetragonoloba*) varieties at germination and early stages. Leg. Res. 5, 23–30.

[B145] Manasa R. Rameshreddy K. Bindumadhava H. Nair R. M. Prasad T. G. Shankar A. G. (2017). Screening mungbean (*Vigna radiata* L.) lines for salinity tolerance using salinity induction response technique at seedling and physiological growth assay at whole plant level. Intl J. Plant Anim. Environ. Sci. 7, 1–12. 10.21276/Ijpae

[B146] Mandhare V. K. Suryawanshi A. V. (2008). Dual resistant against powdery mildew and yellow mosaic virus in greengram. Agric. Sci. Digest. 28, 39–41.

[B147] Manivannan N. Sethuraman K. Natarajan S. (2001). Screening of Greengram (*Vigna radiata* (L.) Wilczek) germplasm for yellow mosaic resistance. Leg. Res. 24, 268–271.

[B148] Mansoor-Ul-Hassan A. R. Akbar R. Latif A. (1998). Varietal response of mung and mash beans to insect attack. Pak. J. Entomol. 20, 43–46.

[B149] Mao Y. B. Cai W. J. Wang J. W. Hong G. J. Tao X. Y. Wang L. J. (2007). Silencing a cotton bollworm P450 monooxygenase gene by plant-mediated RNAi impairs larval tolerance of gossypol. Nat. Biotechnol. 25, 1307–1313. 10.1038/nbt1352 17982444

[B150] Marappa N. (2008). Screening of mungbean genotypes and its wild relatives for resistant sources to Cercospora leaf spot disease. Asian J. Bio. Sci. 3, 324–326.

[B151] Marimuthu G. Rajan S. Chandrashekaran M. K. (1981). Social entrainment of the circadian rhythm in the flight activity of the Microchiropteran bat Hipposideross T) eoris. Behav. Ecol. Sociobiol. 8, 147–150. 10.1007/BF00300827

[B152] Maxwell F. G. Jennings P. R. (1980). Breeding plants resistant to insects. New York: Wiley.

[B153] Mbeyagala K. E. Amayo R. Obuo J. P. Pandey A. K. War A. R. Nair R. M. (2017). A manual for mungbean (greengram) production in Uganda. Natl. Agric. Res Org. (NARO), 32.

[B154] Mei L. Cheng X. Z. Wang S. H. Wang L. X. Liu C. Y. Sun L. (2009). Relationship between bruchid resistance and seed mass in mungbean based on QTL analysis. Genome 52, 589–596. 10.1139/G09-031 19767890

[B155] Mishra S. P. Asthana A. N. Yadav L. (1988). Inheritance of Cercospora leaf spot resistance in mungbean, *Vigna radiata* (L.) Wilczek. Plant Breed. 100, 228–229. 10.1111/j.1439-0523.1988.tb00245.x

[B156] Misra N. Gupta A. K. (2006). Interactive effects of sodium and calcium on proline metabolism in salt tolerant green gram cultivar. Am. J. Plant Physiol. 1, 1–12. 10.3923/ajpp.2006.1.12

[B157] Miyagi M. Humphry M. E. Ma Z. Y. Lambrides C. J. Bateson M. Liu C. J. (2004). Construction of bacterial artificial chromosome libraries and their application in developing PCR-based markers closely linked to a major locus conditioning bruchid resistance in mungbean (*Vigna radiata* L. Wilczek). Theor. Appl. Genet. 110, 151–156. 10.1007/s00122-004-1821-7 15490104

[B158] Moe K. T. Chung J.-W. Cho Y.-I. Moon J.-K. Ku J.-H. Jung J.-K. (2011). Sequence information on simple sequence repeats and single nucleotide polymorphisms through transcriptome analysis of mungbean. J. Integr. Plant Biol. 53, 63–73. 10.1111/j.1744-7909.2010.01012.x 21205180

[B159] Moghadam M. B. Vazan S. Darvishi B. Golzardi F. Farahani M. E. (2011). Effect of mungbean (*Vigna radiate*) living mulch on density and dry weight of weeds in corn (Zea mays) field. Commun. Agric. Appl. Biol. Sci. 76, 555–559. 22696966

[B160] Mondol M. E. A. Rahman H. Rashid M. H. Hossain M. A. Islam M. M. (2013). Screening of mungbean germplasm for resistance to mungbean yellow mosaic virus. Int. J. Sustain. Crop Prod. 8, 11–15.

[B161] Moradi A. Ahmadi A. Hoseinzadeh A. (2009). Agronomic and Physiological interaction of Mung bean (Partov) to sever and light stress in different stages. J. Agric. Res. 12, 659–671.

[B162] Muhammad A. K. Sajjad H. Yasir A. (2018). Evaluation of mung bean germplasm for resistance against mung bean yellow mosaic virus and whitefly population in relation to epidemiological factors. Agric. Res. Tech: Open Access J. 18, 556058. 10.19080/ARTOAJ.2018.18.556058

[B163] Munawar M. H. Iqbal S. M. Malik S. R. Chattha M. R. Ali A. (2011). Identification of resistant sources in mungbean to bacterial leaf spot disease. Mycopath 9, 71–72.

[B164] Munawar M. H. Ali A. Malik S. R. (2014). Identification of resistance in mungbean and mashbean germplasm against mungbean yellow mosaic virus Pakistan. J. Agric. Res. 27, 129–135.

[B165] Murray J. D. Michaels T. E. Cardona C. Schaafsma A. W. Pauls K. P. (2004). Quantitative trait loci for leafhopper (*Empoasca fabae* and *Empoasca kraemeri*) resistance and seed weight in the common bean. Plant Breed. 123, 474–479. 10.1111/j.1439-0523.2004.01020.x

[B166] Nadeem M. Li J. Yahya M. Sher A. Ma. C. Wang X. (2019). Research progress and perspective on drought stress in legumes: a review. I. J. Mol. Sci. 20, 1–32. 10.3390/ijms20102541 PMC6567229 31126133

[B167] Nadeem S. Hamed M. Asghar M. J. Abbas G. Saeed N. A. (2014). Screening of mungbean (*Vigna radiata* (L.) Wilczek) genotypes against sucking insect-pests under natural field conditions. Pak. J. Zool. 46, 863–866.

[B168] Naher N. Alam A. K. (2010). Germination, growth and nodulation of mungbean (*Vignaradiata* L.) as affected by sodium chloride. Int. J. Sustain. Crop Prod. 5, 8–11.

[B169] Nair R. M. Götz M. Winter S. Giri R. R. Boddepalli V. N. Sirari A. (2017). Identification of mungbean lines with tolerance or resistance to yellow mosaic in fields in India where different begomovirus species and different *Bemisia tabaci* cryptic species predominate. Eur. J. Plant Path. 149, 349–365. 10.1007/s10658-017-1187-8

[B170] Noble T. Young A. Douglas C. Williams B. Mundree S. (2019). Diagnosis and management of halo blight in Australian mungbeans: a review. Crop Pasture Sci. 70, 195–203. 10.1071/CP18541

[B171] Noble T. J. Tao Y. Mace E. S. Williams B. Jordan D. R. Douglas C. A. (2018). Characterization of linkage disequilibrium and population structure in a mungbean diversity panel. Front. Plant Sci. 8, 2102. 10.3389/fpls.2017.02102 29375590 PMC5770403

[B172] Oghiakhe S. Jackai L. E. N. Makanjuola W. A. (1992). A rapid visual field screening technique for resistance of cowpea (Vigna unguiculata) to the legume pod borer *Maruca testulalis* (Lepidoptera: Pyralidae). Bull. Entomol. Res. 82, 507–512. 10.1017/S0007485300042589

[B173] Omo-Ikerodah E. E. Fawole I. Fatokun C. A. (2008). Genetic mapping of quantitative trait loci (QTLs) with effects on resistance to flower bud thrips (*Megalurothrips sjostedti*) identified in recombinant inbred lines of cowpea (*Vigna unguiculata* (L.) Walp). Afr. J. Biotechnol. 7, 263–270.

[B174] Osdaghi E. (2014). Occurrence of common bacterial blight on mungbean (*Vigna radiata*) in Iran caused by *Xanthomonas axonopodis* pv. . New Dis. Rep. 30, 9. 10.5197/j.2044-0588.2014.030.009

[B175] Pal S. S. Dhaliwal H. S. Bains S. S. (1991). Inheritance of resistance to yellow mosaic virus in some *Vigna* species. Plant Breed. 106, 168–171. 10.1111/j.1439-0523.1991.tb00496.x

[B176] Pandey A. K. Burlakoti R. R. Kenyon L. Nair R. M. (2018). Perspectives and challenges for sustainable management of fungal diseases of mungbean [*Vigna radiata* (L.) R. Wilczek var. *radiata*]: A Review. Front. Environ. Sci. 6, 53. 10.3389/fenvs.2018.00053

[B177] Pandiyan M. Subbalakshmi B. Alice D. Marimuthu R. (2007). Screening of Mungbean [*Vigna radiata* (L.) Wilczek] germplasm for mungbean yellow mosaic virus. Plant Arch. 7, 375–376.

[B178] Panduranga G. S. Vijayalakshmi K. Loka R. K. Rajashekara H. (2011). Evaluation of mungbean germplasm for resistance against whitefly (*Bemisia Tabaci* Genn.) and mungbean yellow mosaic virus (MYMV) disease. Ind. J. Entomol. 73, 338–342.

[B179] Patel M. B. Srivastava K. P. (1990). Field screening of some high yielding genotypes of mungbean, *Vigna radiata* (Linnaeus) Wilczek to whitefly *Bemisia Tabaci* (Gennadius) and yellow mosaic virus (YMV). Ind. J. Entomol. 52, 547–551.

[B180] Patel P. N. Jindal J. K. (1972). Bacterial leaf spot and halo blight disease of mungbean and other legume in India. Ind. Phytopath. 25, 526–529.

[B181] Paul P. C. Biswas M. K. Mandal D. Pal P. (2013). Studies on host resistance of mungbean against mungbean yellow mosaic virus in the agro-ecological condition of lateritic zone of West Bengal. Bioscan 8, 583–587.

[B182] Pratap A. Gupta D. S. Singh B. B. Kumar S. (2013). Development of super early genotypes in greengram (*Vigna radiata* L. Wilczek). Leg. Res. 36, 105–110.

[B183] Pratap A. Gupta S. Basu S. Tomar R. Dubey S. Rathore M. (2019a). “Towards Development of Climate-Smart Mungbean: Challenges and Opportunities,” in *Genomic Designing of Climate Smart Pulse Crops* Ed. Kole C. (New York: Springer Nature). (*In press*). 10.1007/978-3-319-96932-9_5

[B184] Pratap A. Gupta S. Nair R. M. Gupta S. K. Schafleitner R. Basu P. S. (2019b). Using plant phenomics to exploit the gains of genomics. Agronomy 9, 126. 10.3390/agronomy9030126

[B185] Promila K. Kumar S. (2000). *Vigna radiata* seed germination under salinity. Biol. Plant 43, 423–426. 10.1023/A:1026719100256

[B186] Rabie G. H. (2005). Influence of arbuscular mycorrhizal fungi and kinetin on the response mungbean plants to irrigation with seawater. Mycorrhiza 15, 225–230. 10.1007/s00572-004-0345-y 15765207

[B187] Raguchander T. Prabakar K. Samiyappan R. (2005). Field evaluation of *Pseudomonas fluorescens* and *Bacillus subtilis* on the management of *Cercospora* leaf spot and powdery mildew in urdbean. Leg Res- An Int. J. 28, 137–139.

[B188] Raina S. K. Govindasamy V. Kumar M. Singh A. K. Rane J. Minhas. P. S. (2016). Genetic variation in physiological responses of mungbeans (*Vigna radiata* (L.) Wilczek) to drought. Acta Physiol. Plant 38, 263. 10.1007/s11738-016-2280-x

[B189] Ramakrishnan C. K. D. Savithramma D. L. (2014). Screening of mungbean germplasm for powdery mildew disease Resistant. Int. J. Agron. Agric. Res. 4, 16–21.

[B190] Reddy K. S. (2009a). “A new mutant for yellow mosaic virus resistance in mungbean (*Vigna radiata* L Wilczek) variety SML-668 by recurrent gamma-ray irradiation,” in Induced Plant Mutation in the Genomics Era. Ed. Shu Q. Y. (Rome: Food and Agriculture Organization of the United Nations), 361–362.

[B191] Reddy K. S. (2009b). Identification and inheritance of a new gene for powdery mildew resistance in mungbean (*Vigna radiate* L. Wilczek). Plant Breed. 128, 521–523. 10.1111/j.1439-0523.2008.01609.x

[B192] Reddy K. S. Pawar S. E. Bhatia C. R. (1994). Inheritance of powdery mildew (*Erysiphe polygoni* D.C.) resistance in mungbean (*Vigna radiata* L. Wilczek). Theor. Appl. Genet. 88, 945–948. 10.1007/BF00220800 24186246

[B193] Rodrigues F. A. Marcolino J. Carvalho J. F. C. Nascimento L. C. Neumaier N. Farias J. R. B. (2012). Using subtractive libraries to prospect differentially expressed genes in soybean plants submitted to water deficit. Genet. Mol. Biol. 35, 304–314. 10.1590/S1415-47572012000200011 22802715 PMC3392882

[B194] Rosenzweig C. Elliott J. Deryng D. Ruane A. C. Müller C. Arneth A. (2014). Assessing agricultural risks of climate change in the 21st century in a global gridded crop model inter-comparison. Proc. Natl. Acad. Sci. 111, 3268–3273. 10.1073/pnas.1222463110 24344314 PMC3948251

[B195] Ryley M. J. Tatnell J. R. (2011). Management of the major foliar diseases of mungbeans and peanuts in Australia. In: 4th Asian Conference on Plant Pathology and the 18th Biennial Australasian Plant Pathology Society Conference (ACPP/APPS 2011): New Frontiers in Plant Pathology for Asia and Oceania, 26–29. Darwin, Australia.

[B196] Sadasivan R. Natrajaratnam N. Dabu R. Muralidharan V. Rangasmay S. R. (1988). Response of mungbean cultivars to soil moisture stress at different growth phases. Mungbean Proceeding of the Second International Symposium. AVRCD. Pp.260–262.

[B197] Saha P. Chatterjee P. Biswas A. K. (2010). NaCl pre-treatment alleviates salt stress by enhancement of antioxidant defence and osmolyte accumulation in mungbean (*Vigna radiata* L. Wilczek). Ind. J. Exp. Biol. 48, 593–600. 20882762

[B198] Sahoo B. K. Hota A. K. (1991). Field screening of greengram germplasm against insect pest and disease complex. Madras Agric. J. 78, 84–86.

[B199] Sahoo B. K. Sontakhe B. K. Ruth L. K. (1989). Varietal susceptibility of different greengram and blackgram cultivars to the leaf beetles and pod borer complex. Environ. Ecol. 7, 345–347.

[B200] Salam S. A. Patil M. S. Salimath P. M. (2009). Evaluation of mungbean cultures against MYMV in Karnataka under natural conditions. Leg. Res. 32, 286–289.

[B201] Saleem M. Haris W. A. A. Malik I. A. (1998). Inheritance of yellow mosaic virus in mungbean (*Vigna radiata* L. Wilczek). Pak. J. Phytopath. 10, 30–32.

[B202] Sandhu T. S. Brar J. S. Sandhu S. S. Verma M. M. (1985). Inheritance of resistance to mungbean yellow mosaic virus in greengram. J. Res. Punjab Agric. Univ. 22, 607–611.

[B203] Sandhya Rani C. Eshwari K. B. Sudarshanam A. (2008). Field screening of greengram (*Vigna radiata* L.) entries against thrips (*Thrips palmi*) and spotted pod borer (*Maruca vitrata*). J. Res. ANGRAU 36, 17– 22.

[B204] Sandhya Rani C. Rao G. R. Chalam M. S. V. Kumar P. A. Rao V. S. (2015). Estimation of avoidable losses in mungbean genotypes evaluated under field conditions during summer against *Maruca vitrata*. Int. Res. J. Biol. Sci. 4, 47–54.

[B205] Sandhya Rani C. Rao G. R. Chalam M. S. V. Kumar P. A. Rao V. S. (2014). Field screening of greengram genotypes against *Maruca vitrata* in Summer. J. Agri. Crop Sci. 1, 18–25.

[B206] Sarkar S. Ghosh S. Chatterjee M. Kalita P. D. Lahari T. Maji A. (2011). Molecular markers linked with bruchid resistance in *Vigna radiata* var. sublobata and their validation. J. Plant Biochem. Biotech. 20, 155–160. 10.1007/s13562-011-0039-4

[B207] Schafleitner R. Huang S. M. Chu S. H. Yen J. Y. Lin C. Y. Yan M. R. (2016). Identification of single nucleotide polymorphism markers associated with resistance to bruchids (*Callosobruchus* spp.) in wild mungbean (*Vigna radiata* var. *sublobata*) and cultivated *V. radiata* through genotyping by sequencing and quantitative trait locus analysis. BMC Plant Biol. 16, 159. 10.1186/s12870-016-0847-8 27422285 PMC4946214

[B208] Schafleitner R. Nair R. M. Rathore A. Wang Y. W. Lin C. Y. Chu S. H. (2015). The AVRDC - The World Vegetable Center mungbean (*Vigna radiata*) core and mini core collections. BMC Genomics 16, 344. 10.1186/s12864-015-1556-7 25925106 PMC4422537

[B209] Sehgal A. Sita A. Kadambot H. M. S. Kumar Rakesh Sailaja B. Varshney R. K. (2018). Drought or/and Heat-Stress Effects on Seed Filling in Food Crops: Impacts on Functional Biochemistry, Seed Yields, and Nutritional Quality. Front. Plant Sci. 9, 1–19. 10.3389/fpls.2018.01705 30542357 PMC6277783

[B210] Sehrawat N. Bhat K. V. Sairam R. K. Jaiwal P. K. (2013). Screening of mungbean [*Vigna radiata* (L.) Wilczek] genotypes for salt tolerance. Int. J. Plant. Anim. Environ. Sci 4, 36–43.

[B211] Sehrawat N. Bhat K. V. Kaga A. Tomooka N. Yadav M. Jaiwal P. K. (2014). Development of new gene-specific markers associated with salt tolerance for mungbean (*Vigna radiata* L.Wilczek). Spanish J Agric Res 12 (3), 732–741. 10.5424/sjar/2014123-4843

[B212] Sekar S. Nalini R. (2017). Varietal Screening of Mungbean Genotypes against Whitefly (Bemisia tabaci Genn.), Mungbean Yellow Mosaic Virus (MYMV) and Cercospora leaf Spot. Int. J. Curr. Microbial. App. Sci. 6 1278–1285. 10.20546/ijcmas.2017.603.147

[B213] Senthil-Kumar M. Mysore K. S. (2010) RNAi in Plants: recent developments and applications in agriculture, In: Gene Silencing: Theory, Techniques and Applications Eds. Catalano A. J. Nova Science Publishers, Inc, New York USA pp. 183–199.

[B214] Setter T. L. Waters I. Sharma S. K. Singh K. N. Kulshreshtha N. Yaduvanshi N. P. S. (2009). Review of wheat improvement for waterlogging tolerance in Australia and India: the importance of anaerobiosis and element toxicities associated with different soils. Ann. Bot. 103, 221–235. 10.1093/aob/mcn137 18708642 PMC2707304

[B215] Shad N. Mughal S. M. Farooq K. Bashir M. (2006). Evaluation of mungbean germplasm for resistance against mungbean yellow mosaic begomovirus. Pak. J. Bot. 38, 449–457.

[B216] Shakeel S. Mansoor S. (2012). Salicylic acid prevents the damaging action of salt in mungbean [(*Vigna radiata* L.) Wilczek] seedlings. Pak. J. Bot. 44, 559–562.

[B217] Shanmugasundaram S. (2007). Exploit mungbean with value added products. Acta Hortic. 752, 99–102. 10.17660/ActaHortic.2007.752.12

[B218] Sharma H. C. Saxena K. B. Bhagwat V. R. (1999). “The legume pod borer, *Maruca vitrata*: Bionomics and management,” in Information Bulletin 55 (Patancheru, Andhra Pradesh, India: International Crops Research Institute for the Semi-Arid Tropics).

[B219] Sharma H. C. Ahmad R. Ujagir R. (2005). “Host plant resistance to cotton bollworm/legume pod borer, *Helicoverpa armigera*.,” in Strategies for Heliothis/Helicoverpa management: emerging trends and strategies for future research. Ed. Sharma H. C. (New Delhi: Oxford and IBH), 167–208.

[B220] Sharma L. Priya M. Bindumadhava H. Nair R. M. Nayyar H. (2016). Influence of high temperature stress on growth, phenology and yield performance of mungbean (*Vigna radiata* (L.) Wilczek) under managed growth conditions. Sci. Hort. 213, 379–391. 10.1016/j.scienta.2016.10.033

[B221] Shi A. Chen P. Li D. X. Zheng C. Zhang B. Hou A. (2009). Pyramiding multiple genes for resistance to soybean mosaic virus in soybean using molecular markers. Mol. Breed. 23, 113–124. 10.1007/s11032-008-9219-x

[B222] Shukla V. Baghel S. Maravi K. Singh S. K. (2014). Yield loss assessment in mungbean [*Vigna radiata* (L.) Wilczek] caused by anthracnose [*Colletotrichum truncatum* (schw.) Andrus and moore]. Bioscan 9, 1233–1235.

[B223] Singh D. P. (1997). Tailoring the plant type in pulse crops. Plant Breed. 67, 1213–1220.

[B224] Singh D. P. Singh B. B. (2011). Breeding for tolerance to abiotic stresses in mungbean. J Food Leg. 24, 83–90.

[B225] Singh B. R. Chandra S. Ram S. (2000). Evaluation of mungbean varieties against yellow mosaic virus. Ann. Plant Prot Sci 8, 270–271.

[B226] Singh G. Sharma Y. R. Shanmugasundaram S. Shih S. L. Green S. K. (2004). Improving income and nutrition by incorporating mungbean in cereal fallows in the Indo-Gangetic Plains of South Asia DFID Mungbean Project for 2002-2004. in Proceedings of the Final Workshop and Planning Meeting, Status of Mung Bean Yellow Mosaic Virus Resistance Breeding Ludhiana: Punjab Agricultural University, 27–31 May 2004, 204–213.

[B227] Singh G. Singh S. Sheoran O. P. (2013b). Inheritance of mungbean yellow mosaic virus (mymv) resistance in mungbean [*Vigna radiata* (l.) wilczek]. Leg. Res: An Int. J. 36, 131–137.

[B228] Singh J. Mishra K. K. Singh A. K. (2013a). Current status of web blight of mungbean. Asian J. Soil Sci. 8, 495–504.

[B229] Singh S. P. Singh S. K. (2014). Sources of resistant in mungbean for *Cercospora* leaf spot diseases. Ann. Agric. Biosci. Res. 2, 280–281.

[B230] Singh S. P. Terán H. Muñoz C. G. Takegami J. C. (1999). Two cycles of recurrent selection for seed yield in common bean. Crop Sci. 39, 391–397. 10.2135/cropsci1999.0011183X0039000200015x

[B231] Somta C. Somta P. Tomooka N. Ooi P. A. C. Vaughan D. A. Srinives P. (2008). Characterization of new sources of mungbean (*Vigna radiata* (L.) Wilczek) resistance to bruchids, *Callosobruchus* spp. (Coleoptera: Bruchidae). J. Stored Prod. Res. 44, 316–321. 10.1016/j.jspr.2008.04.002

[B232] Somta P. Ammaranan C. Ooi P. A. C. Srinives P. (2007). Inheritance of seed resistance to bruchids in cultivated mungbean (*Vigna radiata* L.Wilczek). Euphytica 155, 47–55. 10.1007/s10681-006-9299-9

[B233] Sorajjapinun W. Rewthongchum S. Koizumi M. Srinives P. (2005). Quantitative inheritance of resistance to powdery mildew disease in mungbean (*Vigna radiata* (L.) Wilczek). SABRAO J. Breed. Genet. 37, 91–96.

[B234] Soundararajan R. P. Chitra N. Ramasamy M. (2010). Host Plant Resistance to insect-pests of urdbean and mungbean. In: National workshop on paradigm shifts in research on crop resistance to pests, Annamalai University, Annamalai Nagar, March 4–5 Mar, p 57–58.

[B235] Southgate B. J. (1979). Biology of the bruchidae. Annu. Rev. Entomol. 24, 449–473. 10.1146/annurev.en.24.010179.002313

[B236] Sudha M. Karthikeyan A. Anusuya P. Ganesh N. M. Pandiyan M. Senthil N. (2013). Inheritance of resistance to mungbean yellow mosaic virus (MYMV) in inter and intra specific crosses of mungbean (*Vigna radiata*). Am. J. Plant. Sci. 4, 1924–1927. 10.4236/ajps.2013.410236

[B237] Sujatha K. Kajjidoni S. T. Patil P. V. Somashekhar G. (2011). Heterosis for productivity related traits involving diverse parents for powdery mildew reaction in mungbean. J. Food Leg. 24, 101–105.

[B238] Suman S. Sharma S. K. Kumar H. Shahi V. K. (2015). Screening of mungbean [*Vigna radiata* (L.) Wilczek] genotypes for resistance to mungbean yellow mosaic virus (MYMV). Environ. Ecol. 33, 855–859.

[B239] Sun S. Zhi Y. Zhu Z. Jin J. Duan C. Wu X. (2017). An emerging disease caused by *Pseudomonas syringae* pv. *phaseolicola* Threatens mungbean production in China. Plant Dis. 101, 95–102. 10.1094/PDIS-04-16-0448-RE 30682319

[B240] Sunkar R. Zhu J. K. (2004). Novel and stress-regulated micro RNAs and other small RNAs from Arabidopsis. Plant Cell 16, 2001–2019. 10.1105/tpc.104.022830 15258262 PMC519194

[B241] Suraninpong P. Introduction and expression of cholesterol oxidase gene in a bacterium [Escherichia coli M15 (pREP4)] and mungbean [Vigna radiata (L.) Wilczek]. p. 162, 2002, PhD Thesis, Suranare University of Technol,.

[B242] Swaminathan R. Singh K. Nepalia V. (2012). “Insect-pests of green gram *Vigna radiata* (L.) Wilczek and their management,”. In Agriculture Science Ed. Aflakpui G (Agricultural Science, India: Intech Publishers), 197–222. 10.5772/35176

[B243] Swarnalatha P. (2007). Germplasm screening and insecticidal management of pest complex in greengram (Vigna radiata (L.) Wilczek). M.Sc.(Ag.) Thesis. Rajendranagar, Hyderabad: Acharya NG Ranga Agricultural University.

[B244] Swathi L. Reddy D. M. Sudhakar P. Vineela V. (2017). Screening of Mungbean (*Vigna radiata* L. Wilczek) genotypes against water stress mediated through polyethylene glycol. Int. J. Curr. Microbiol. App. Sci. 6, 2524–2531. 10.20546/ijcmas.2017.610.296

[B245] Taggar G. K. Gill R. S. (2012). Preference of whitefly, *Bemisia tabaci*, towards pi genotypes: role of morphological leaf characteristics. Phytoparasitica 40, 461–474. 10.1007/s12600-012-0247-z

[B246] Talekar N. S. Lin Y. H. (1981). Two sources with differing modes of resistance to *Callosobruchus chinensis* in mungbean. J. Econ. Entomol. 74, 639–642. 10.1093/jee/74.5.639

[B247] Talekar N. S. Lin Y. H. (1992). Characterization of *Callosobruchus chinensis* resistance in mungbean. J. Econ. Entomol. 85, 1150–1153. 10.1093/jee/85.4.1150

[B248] Talekar N. S. (1990). Agromyzid flies of food legumes in the tropics. New Delhi: Wiley Eastern Limited, 299.

[B249] Taylor J. D. Teverson D. M. Allen M. A. Pastor-Corrales M. A. (1996). Identification and origin of races of Pseudomonas syringae pv. phaseolicola from Africa and other bean growing areas. Plant Pathol. 45, 469–478. 10.1046/j.1365-3059.1996.d01-147.x

[B250] Tazeen S. Mirza B. (2004). Factors affecting *Agrobacterium tumefaciens* mediated genetic transformation of *Vigna radiata* (l.) Wilczek. Pak. J. Bot. 36 (4), 887–896.

[B251] Terán H. Singh S. P. (2010). Recurrent selection for physiological resistance to white mold in dry bean. Plant Breed. 129 327–333. 10.1111/j.1439-0523.2009.01679.x

[B252] Thakur R. P. Patel P. N. Verma J. P. (1977). Genetical relationships between reactions to bacterial leaf spot, yellow mosaic and Cercospora leaf spot diseases in mungbean (*Vigna radiata*). Euphytica 26, 765–774. 10.1007/BF00021705

[B253] Thi T. O. Aung K. Myint T. (2005). Natural incidence of the bean stem fly Ophiomyia phaseoli (Tryon) (Diptera: Agromyzidae) in different plant growth stages of greengram in different growing seasons. Proceedings of the Fourth Agricultural Research Conference in Myanmar pp135–143.

[B254] Thomas M. Robertson J. Fukai S. Peoples M. B. (2004). The effect of timing and severity of water deficit on growth development, yield accumulation and nitrogen fixation of mung bean. Field Crops Res. 86, 67–68. 10.1016/S0378-4290(03)00120-5

[B255] Toker C. Mutlu N. (2011). “Breeding for abiotic stress,” in Biology and Breeding of Food Legumes. Eds. Pratap A. Kumar J. (CAB International) Wallingford, UK, 241–260. 10.1079/9781845937669.0241

[B256] Tomooka N. Kashiwaba K. Vaughan D. Ishimoto M. Egawa Y. (2000). The effectiveness of evaluating wild species, searching for sources of resistance to bruchid beetle in the genus *Vigna* sub species *Ceratotropis*. Euphytica 115, 27–41. 10.1023/A:1003906715119

[B257] Tomooka N. Lairungruang C. Nakeeraks P. Egawa. Y. Thavarasook C. (1992). Development of bruchid resistant mungbean using wild mungbean germplasm in Thailand. Plant Breed. 109, 60–66. 10.1111/j.1439-0523.1992.tb00151.x

[B258] Tripathy S. Mohanty P. Jena M. Dash S. Lenka D. Mishra D. (2016). Identification of seed storage protein markers for drought tolerance in mungbean. Res. Biotechnol. 7, 3–11. 10.19071/rib.2016.v7.2895

[B259] Villareal J. M. Hautea D. M. Carpena A. L. (1998). Molecular mapping of the bruchid resistance gene in mungbean *Vigna radiata* L. Philippine J. Crop Sci. 23 (1), 1–9.

[B260] Wang L. Wu C. Zhong M. Zhao D. Mei L. Chen H. (2016). Construction of an integrated map and location of a bruchid resistance gene in mung bean. Crop J. 4, 360–366. 10.1016/j.cj.2016.06.010

[B261] Wang L. F. Jing W. U. Jing R. L. Cheng X. Z. Wang S. M. (2015). Drought resistance identification of mungbean germplasm resources at seedlings stage. Acta Agron. Sin. 41, 145–153. 10.3724/SP.J.1006.2015.00145

[B262] Wang L. F. Jing W. U. Jing R. L. Cheng X. Z. Wang S. M. (2014). Drought resistance identification of mungbean germplasm resources at bud stage. J. Plant Genet. Resour. 15, 498–503.

[B263] War A. R. Murugesan S. Boddepalli V. N. Srinivasan R. Nair R. M. (2017). Mechanism of Resistance in Mungbean [*Vigna radiata* (L.) R. Wilczek var. *radiata*] to Bruchids, *Callosobruchus* spp. (Coleoptera: Bruchidae). Front. Plant Sci. 8, 1031. 10.3389/fpls.2017.01031 28676807 PMC5477293

[B264] Watanasit A. Pichitporn S. (1996). Improvement of mungbeanfor resistance to bruchids. In: Srinives P Kitbamroong C Miyazaki S (eds) Mungbean germplasm: collection, evaluation and utilization for breeding program. Japan International Research Center for Agricultural Sciences, Tsukuba, Japan, pp 67–71.

[B265] Watanasit A. Ngampongsai S. Thanomsub W. (2001). “The use of induced mutations for mungbean improvement. Report of an FAO/IAEA Seminar on Mutation Techniques and Molecular Genetics for Tropical and Subtropical Plant Improvement in Asia and the Pacific Region. October 11-15, 1999,” in The Philippines, 11–12.

[B266] Wesley S. V. Helliwell C. A. Smith N. A. Wang M. B. Rouse D. T. Liu Q. (2001). Construct design for efficient, effective and high-throughput gene silencing in plants. Plant J. 27, 581–590. 10.1046/j.1365-313X.2001.01105.x 11576441

[B267] Wongpiyasatid A. Chotechuen S. Hormchan P. Ngampongsai S. Promcham W. (2000). Induced mutations in mungbean breeding: regional yield trial of mungbean mutant lines. Kasetsart J. (Nat. Sci.) 34, 443– 449.

[B268] Wongpiyasatid A. Chotechuen S. Hormchan P. Srihuttagum M. (1999). Evaluation of yield and resistance to powdery mildew, Cercospora leaf spot and cowpea weevil in mungbean mutant lines. Kasetsart J. (Nat. Sci.) 33, 204–215.

[B269] Xiong X. P. Kurthkoti K. Chang K. Y. Lichinchi G. Nabanita De Schneemann A. (2013). Core small nuclear ribonucleoprotein particle splicing factor SmD1 modulates RNA interference in Drosophila. Proc. Natl. Acad. Sci. USA 110 (41), 16520–16525. 10.1073/pnas.1315803110 24067655 PMC3799365

[B270] Yadav D. L. Jaisani P. Pandey R. N. (2014a). Identification of sources of resistant in mungbean genotypes and influence of fungicidal application to powdery mildew epidemics. Int. J. Curr. Microbiol. Appl. Sci. 3, 513–519.

[B271] Yadav D. L. Pandey R. N. Jaisani P. Gohel N. M. (2014b). Sources of resistant in mungbean genotypes to Cercospora leaf spot disease and its management. Afr. J. Agric. Res. 9, 3111–3114. 10.5897/AJAR2014.8860

[B272] Yadav G. S. Dahiya B. (2004). Performance of mungbean genotypes against whitefly and yellow mosaic. Ann. Biol. 20, 57–59.

[B273] Yao Y. Cheng X. Ren G. (2015). A 90-day study of three bruchid-resistant mungbean cultivars in Sprague–Dawley rats. Food Chem. Toxicol. 76, 80–85. 10.1016/j.fct.2014.11.024 25533792

[B274] Ye H. Liu S. Tang B. Chen J. Xie Z. Nolan T. M. (2017). RD26 mediates crosstalk between drought and brassinosteroid signalling pathways. Nat. Commun. 8, 14573. 10.1038/ncomms14573 28233777 PMC5333127

[B275] Young N. D. Danesh D. Menancio-Hautea D. Kumar L. (1993). Mapping oligogenic resistance to powdery mildew in mungbean with RFLPs. Theor. Appl. Genet. 87, 243–249. 10.1007/BF00223772 24190220

[B276] Young N. D. Kumar L. Menancio-Hautea D. Danesh D. Talekar N. S. Shanmugasundarum S. (1992). Mapping of a major bruchid resistance gene in mungbean (*Vigna radiata*, L Wilczek). Theor. Appl. Genet. 84, 839–844. 10.1007/BF00227394 24201484

[B277] Zahid M. A. Islam M. M. Begum M. R. (2008). Determination of economic injury levels of Maruca vitrata in Green gram. J. Agric. Rural Dev. 6, 91–97. 10.3329/jard.v6i1.1662

[B278] Zha W. Peng X. Chen R. Du B. Zhu L. He G. (2011). Knockdown of midgut genes by dsRNAtransgenic plant-mediated RNA interference in the Hemipteran insect *Nilaparvata lugens*. PLoS One 6, e20504. 10.1371/journal.pone.0020504 21655219 PMC3105074

[B279] Zhimo V. Y. Panja B. N. Saha J. Nath R. (2013). Evaluation of mungbean genotypes for resistance against Cercospora leaf spot and Yellow Mosaic disease under field condition. J. Mycopathol. Res. 51, 273–278.

